# Developing a Tailored eHealth Self-Management Intervention for Patients With Chronic Kidney Disease in China: Intervention Mapping Approach

**DOI:** 10.2196/48605

**Published:** 2024-06-13

**Authors:** Hongxia Shen, Rianne van der Kleij, Paul J M van der Boog, Niels H Chavannes

**Affiliations:** 1 School of Nursing, Guangzhou Medical University Guangzhou China; 2 Department of Public Health and Primary Care, Leiden University Medical Centre Leiden Netherlands; 3 National eHealth Living Lab, Leiden University Medical Centre Leiden Netherlands; 4 Department of Nephrology, Leiden University Medical Centre Leiden Netherlands

**Keywords:** eHealth, self-management, intervention mapping, chronic kidney disease, intervention development, mobile phone

## Abstract

**Background:**

Chronic kidney disease (CKD) is a major public health concern. Adequate self-management skills are vital to reduce CKD burden, optimize patient health outcomes, and control health care expenditures. Using eHealth to support CKD self-management has the potential to promote healthy behaviors and improve health outcomes of patients with CKD. However, knowledge of the implementation of such interventions in general, and in China specifically, is still limited.

**Objective:**

This study aims to develop a tailored eHealth self-management intervention for patients with CKD in China based on the Dutch Medical Dashboard (MD) eHealth self-management intervention.

**Methods:**

We used an intervention mapping approach. In phase 1, a systematic review and 2 qualitative studies were conducted to examine the needs, beliefs, and perceptions of patients with CKD and health care professionals regarding CKD self-management and eHealth interventions. Afterward, key factors gathered from the aforementioned studies were categorized following the 5 domains of the Consolidated Framework for Implementation Research (CFIR). In phase 2, we specified program outcomes, performance objectives, determinants, theory-based methods, and practical strategies. Knowledge obtained from previous results was combined to complement core components of the MD self-management intervention and adapt them for Chinese patients with CKD. Additionally, the CFIR–Expert Recommendations for Implementing Change Matching Tool was pragmatically used to generate a list of potential implementation strategies to address the key factors influencing the implementation of eHealth CKD self-management interventions, and implementation strategies were discussed and finalized with the intervention monitoring group.

**Results:**

An overview of the CFIR domains showed the essential factors influencing the implementation of eHealth CKD self-management interventions in Chinese settings, including “knowledge and beliefs” in the domain “individual characteristics,” “quality and advantage of eHealth intervention” in the domain “intervention characteristics,” “compatibility” in the domain “inner setting,” and “cultural context” in the domain “outer setting.” To ensure the effectiveness of the Dutch MD–based self-management intervention, we did not change the core self-management intervention components of MD that underlie its effectiveness, such as self-monitoring. We identified surface-level cultural adaptations involving customizing intervention content, messages, and approaches to the observable cultural characteristics of the local population to enhance the intervention’s appeal, receptivity, and feasibility, such as providing video or voice call options to support interactions with health care professionals. Furthermore, the adapted modules such as *Knowledge Center* and *My Self-Monitoring* were developed in a mobile health app.

**Conclusions:**

Our study resulted in the delivery of a culturally tailored, standardized eHealth self-management intervention for patients with CKD in China that has the potential to optimize patients’ self-management skills and improve health status and quality of life. Moreover, our study’s research approach and results can inform future research on the tailoring and translation of evidence-based, eHealth self-management interventions to various contexts.

**Trial Registration:**

ClinicalTrials.gov NCT04212923; https://classic.clinicaltrials.gov/ct2/show/NCT04212923

## Introduction

### Background

Chronic kidney disease (CKD) is a serious public health problem [1-5]. The burden of CKD is highest in low- and middle-income countries [6,7]. In China, around 132 million patients are affected by CKD—accounting for one-fifth of the global burden of CKD [8]. Interventions supporting self-management can improve patient self-management behaviors [9-11] and health outcomes [12] and even slow down disease progression [13-15]. Optimizing CKD self-management is vital to reduce disease burden and control health care expenditures [13].

eHealth-based interventions are being developed to support CKD self-management [16-18]. There is previous evidence suggesting that eHealth self-management interventions can promote healthy behaviors and improve the health outcomes of patients with CKD [19-21] and are feasible and acceptable for patients with CKD and health care professionals (HCPs) [22]. An example of a rigorously studied and effective eHealth CKD self-management intervention is Medical Dashboard (MD) [23-25]. MD was developed in the Netherlands, enabling patients and HCPs to monitor and track healthy behaviors and disease parameters. This platform has been used in the Kidney Diseases and Transplantation Outpatient Clinic at Leiden University Medical Center since February 2016. In a randomized controlled trial, the use of MD effectively improved patients’ adherence to sodium intake restriction and blood pressure control [23]. In addition, patients and HCPs were satisfied with the MD self-management intervention [24].

Improving CKD self-management using digital innovations has been named a top priority in China [26,27]. Policy makers and health care experts in China launched the national health strategy *Healthy China 2030* [28]. This strategy describes eHealth technology as an essential pillar to improve disease self-management and the accessibility and cost-effectiveness of care in (rural) China—where >558 million people have access to a mobile phone. In rural areas in China, an estimated 8.6% of adults have CKD [29]. There is a lack of a strong primary care system in rural China to provide adequate health care for patients with CKD; most of the high-quality resources in medical care such as human capital and modern diagnostic and therapeutic technologies are concentrated in Chinese hospitals. The long distance to health care facilities is a substantial problem for patients with CKD in rural areas. eHealth interventions provide great potential to address these challenges, such as so-called “internet hospitals” that allow patients to receive high-quality care from a top-tier hospital from either their own home or a local clinic through a video or telephone connection [30]. In addition, patients with CKD and HCPs in China feel highly positive toward using eHealth to support CKD self-management [31]. Thus, there is a high need and significant momentum for implementing effective eHealth-based interventions—such as MD—to support CKD self-management in China.

The implementation of self-management interventions should take into account the patients’ needs regarding interventions or the fact that self-management occurs in a social context [32]. Hence, applying a “one-size-fits-all” approach and simply translating effective CKD self-management interventions to a different context is not sufficient. According to the recently published SETTING (Setting Exploration Treasure Trail to Inform Implementation Strategies) tool, mapping local context characteristics and needs before translating health interventions is essential for successful implementation [33]. By doing so, context-specific, tailored intervention development and adaptation can be performed, ensuring an optimal fit with the local context.

Implementation science provides guidance on assessing and integrating knowledge about local contexts into the eHealth intervention development [34,35]. For instance, the intervention mapping (IM) method [36] ensures a theory-based approach from the recognition of local needs to the identification of tailored intervention and implementation strategies and has been used to develop and adapt evidence-based interventions [37,38]. However, evidence regarding how IM may guide the translation of a complex eHealth self-management intervention for patients with CKD from high-resource to low-resource settings is still limited.

eHealth self-management interventions have the potential to fundamentally improve the quality of life and health outcomes of patients with CKD in China. The MD-based self-management intervention has been researched extensively and proven effective. In addition, it matches the needs identified in our earlier studies among Chinese patients with CKD [31,39]. Moreover, we are in close contact with the developers and, therefore, able to discuss amendments with the team and allowed to upscale a tailored version of the intervention to the Chinese context. Therefore, we aimed to use IM to develop a tailored eHealth self-management intervention for patients with CKD in China based on the Dutch MD intervention (clinical trial registration: ClinicalTrials.gov NCT04212923). Further details on the methodology and design of the extensive study can be found elsewhere [40].

### Objectives

In concordance with IM, this study comprised two parts: (1) *phase 1: needs, beliefs, and perceptions* (step 1 of IM)—examine the needs, beliefs, and perceptions of both patients with CKD and HCPs regarding CKD self-management and related eHealth interventions; and (2) *phase 2: intervention and implementation development and planning* (steps 2-5 of IM)—complement the core components of the MD self-management intervention for patients with CKD and adapt them to the Chinese context.

## Methods

### Overview

IM consists of six steps: (1) a needs assessment, (2) preparation of change objective matrices, (3) selection of theory-informed intervention methods and strategies, (4) development of a tailored intervention, (5) implementation, and (6) evaluation plans. For our study, steps 1 to 5 were completed between 2017 and 2023. The tasks performed to complete each step are described in the following paragraphs.

### Phase 1: Needs, Beliefs, and Perceptions

#### Intervention Monitoring Group

First, an intervention monitoring group including both Dutch and Chinese experts and other key stakeholders was established. This group consisted of 2 researchers, 1 nephrologist, 1 nurse in CKD practice, and 1 implementation specialist. The expert group has ample experience with CKD care and implementing (eHealth) self-management interventions. The intervention monitoring group discussed the progress and the execution of major steps, such as the needs assessment and intervention development.

#### Identify Key Factors Influencing the (Potential) Implementation of eHealth CKD Self-Management Interventions

##### Evidence in Previous Studies

Evidence regarding key factors influencing the implementation of eHealth CKD self-management interventions was gathered from our systematic review [21] and qualitative studies conducted in the Chinese context [31,39].

A systematic review was conducted to summarize the evidence regarding the implementation and effectiveness of eHealth self-management interventions for patients with CKD. A total of 24 articles were included. Details on the review are published elsewhere [21].

In addition, 2 qualitative studies were conducted. The following is a short description of the methods used in the 2 studies. The inclusion and exclusion criteria for patients with CKD are shown in Textbox 1. Further details can be found elsewhere [31,39].

**Eligibility criteria for patients with chronic kidney disease (CKD).**

**Inclusion criteria**

- Patients (1) aged >18 years; (2) with a diagnosis of CKD with markers of kidney damage or a glomerular filtration rate of <60 mL/min/1.73 m^2^ persisting for ≥3 months based on Kidney Disease: Improving Global Outcomes (KDIGO) guidelines; (3) at all CKD stages following the KDIGO staging of CKD (stages G1-G5 and dialysis) including non–kidney transplantation; and (4) who spoke Chinese

**Exclusion criteria**

- Individuals unable to provide written informed consent or use the electronic app due to physical disabilities such as eyesight problems or mental disabilities such as psychosis, personality disorders, or schizophrenia (final decision regarding exclusion to be made by the treating physician)
- Individuals unable to write or read

In the first study, the perceptions and needs of patients with CKD and HCPs regarding CKD self-management in China were examined. A basic interpretive, cross-sectional qualitative study comprising semistructured interviews and observations was conducted with 11 patients with CKD and 10 HCPs in the Department of Nephrology at the First Affiliated Hospital of Zhengzhou University in Henan province, China.

The second qualitative study examined the perceptions, attitudes, and needs of patients with CKD and HCPs regarding eHealth-based (self-management) interventions in general and the Dutch MD intervention specifically. A basic interpretive, cross-sectional qualitative study was conducted comprising semistructured interviews with 11 patients with CKD and 10 HCPs and 2 focus group discussions with 9 patients with CKD in the Department of Nephrology at the First Affiliated Hospital of Zhengzhou University in Henan province, China.

##### Key Factors Identified

The key factors (ie, barriers and facilitators) identified through the review and qualitative studies were gathered and categorized following the 5 domains of the Consolidated Framework for Implementation Research (CFIR) [41,42]. The CFIR provides a pragmatic structure for identifying potential implementation strategies for interventions in health systems at multiple levels [43-47]. It has also been successfully used to identify determinants of behavior change and optimize the design and effectiveness of self-management interventions [48].

Next, the intervention monitoring group decided which factors were the most important and changeable in each domain of the CFIR and summarized key lessons in designing eHealth CKD self-management interventions in the Chinese context.

### Phase 2: Intervention and Implementation Development and Planning (Steps 2-5 of IM)

#### Step 2: Preparing Matrices of Change Objectives

First, we formulated program outcomes [36] based on the socioecological model [49]. Second, we subdivided program outcomes into performance objectives (POs). The POs are the required actions to accomplish change in the behavioral and environmental outcomes [36], which were linked to key determinants identified in the needs assessment [36]. We used the Theoretical Domains Framework to identify and select relevant determinants of behavior [50]. In total, 2 researchers identified and linked the determinants, and discussions resolved discrepancies. In addition, the intervention monitoring group evaluated the selected determinants for relevance and changeability. Finally, based on the determinants identified, we specified change objectives [36].

#### Step 3: Selecting Theory-Informed Intervention Methods and Practical Strategies

The matrices of change objectives guided the selection of theoretical methods and practical intervention strategies [36]. Relevant theoretical methods such as the Theory of Planned Behavior (TPB) [51], the Health Belief Model (HBM) [52], the IM book [36], and the taxonomy of behavior change techniques (BCTs) [53] were identified through a literature review. These theoretical methods or BCTs were translated into practical strategies tailored to each change objective. In addition, the intervention monitoring group discussed the acceptability and feasibility of these practical strategies based on existing CKD self-management interventions and results from the needs assessment.

#### Step 4: Developing a Tailored MD-Based Intervention (Plan)

First, based on the results of steps 1 to 3, the intervention monitoring group formulated guiding principles to complement the MD and adapt it into a tailored self-management intervention for the Chinese context. A logic model was also built to map and structure the causal mechanisms of the intervention by providing detailed evidence and strategies [54,55].

Second, the intervention components were adapted for the Chinese context. To ensure the effectiveness of the self-management intervention, we did not change the core self-management intervention components of MD that underlie its effectiveness. In addition, the intervention monitoring group used the technology functionality framework [56,57] to prioritize eHealth features, app functionalities, and strategies to reduce the complexity and improve the accessibility and usability of the app content. Low-fidelity prototypes [58] were developed to visualize design solutions and determine intervention requirements.

#### Step 5: Developing an Adoption and Implementation Plan

The intervention monitoring group pragmatically used the CFIR–Expert Recommendations for Implementing Change (ERIC) Matching Tool [59] to generate a list of potential implementation strategies. They also discussed the output of the implementation strategies to ensure that these strategies would be best placed to deliver the adapted mobile app for CKD self-management.

### Ethical Considerations

This study has been approved by the ethical board of the Guangzhou Medical University (reference L202212020). All participants gave written informed consent. All data were anonymized. The participants volunteered to participate in this study, and no compensation was provided.

## Results

### Phase 1: Needs, Beliefs, and Perceptions

#### Identification of Key Barriers and Facilitators

##### Evidence in Previous Studies

Our review [21] identified promising intervention components for CKD self-management and related implementation determinants. For instance, *self-monitoring* and *using messages or alerts to nudge patients toward displaying healthy behaviors* were found to optimize patient self-management skills. In addition, the determinant *ability of HCPs to monitor and, if necessary, anticipate patient measurements online* influenced patients’ adherence to interventions. The review results are published elsewhere [21].

Furthermore, the first qualitative study revealed a paternalistic patient-HCP relationship. Barriers, facilitators, and needs regarding CKD self-management were frequently related to (a lack of) knowledge and environmental context and resources. The second qualitative study highlighted factors such as a lack of information, perceived trustworthiness and safety of eHealth interventions, clinical compatibility and complexity of eHealth, time constraints, and eHealth literacy. Suggestions for adapting and implementing the Dutch MD intervention in China focused on improving the functionalities and content of MD. Further details can be found elsewhere [31,39].

##### Key Factors Identified Using a Theoretical Framework

**Figure 1:**
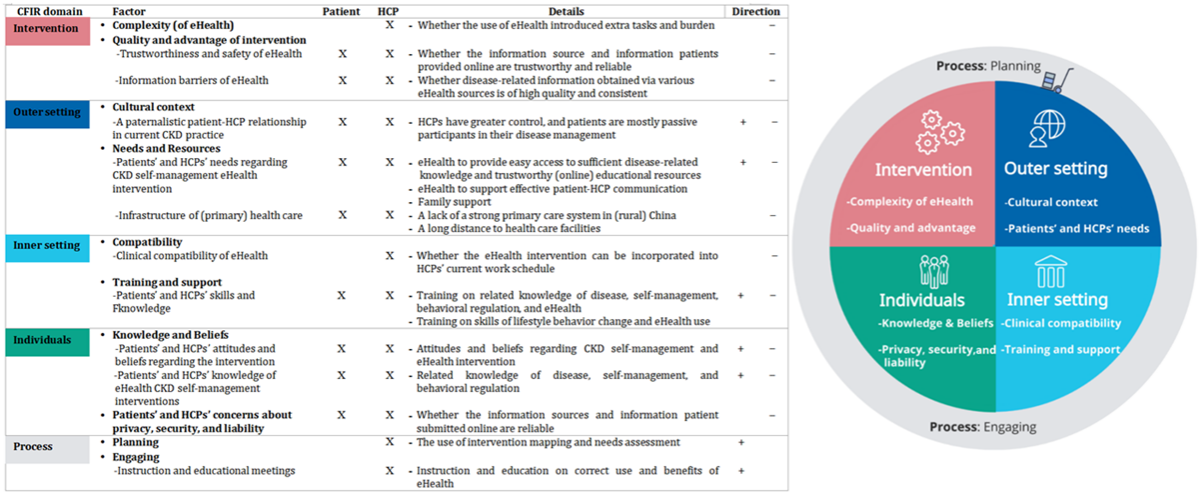
Overview of the different domains of the Consolidated Framework for Implementation Research (CFIR) related to eHealth chronic kidney disease (CKD) self-management intervention implementation in the Chinese context. X: mentioned by stakeholders; +: if the factor is present or considered a facilitator; -: if the factor is present or considered a barrier; HCP: health care professional.

Key factors gathered from the aforementioned studies [21,31,39] were structured and categorized following the 5 domains of the CFIR. Figure 1 provides an overview of the key factors per CFIR domain that facilitate and hinder implementation.

#### Lessons Learned

Several key lessons were summarized for designing eHealth CKD self-management interventions in the Chinese context. For instance, to ensure that the eHealth app is time saving, eHealth functionalities must be simple and easy to use, and eHealth navigation must be clear. Other key lessons are summarized in Multimedia Appendix 1.

### Phase 2: Intervention and Implementation Development and Planning (Steps 2-5 of IM)

#### Step 2: Preparing Matrices of Change Objectives

The program outcomes were specified based on the socioecological model [49] (refer to Textbox 2 for the specific outcomes). In addition, a total of 12 POs and 124 accompanying change objectives were identified (for details, refer to Multimedia Appendix 2). The POs are shown in Textbox 3.

**Specific intervention outcomes by socioecological level.**

**Individual**

- Illness perception
  Patients improve their illness perception (ie, their knowledge and beliefs regarding chronic kidney disease [CKD] symptoms, CKD medical conditions, and health threats).
- Knowledge
  Patients and health care professionals (HCPs) improve their knowledge of CKD self-management.
- Behavior
  Patients improve their CKD self-management behavior tested using a CKD self-management instrument after a 9-month intervention period.
  Patients receive sufficient web-based support and have less face-to-face contact with HCPs.

**Interpersonal**

- Patients receive sufficient family and peer support during the process of CKD self-management.
- Patients receive sufficient web-based support and have less face-to-face contact with HCPs for minor complaints. Therefore, HCPs can have more time to provide care to patients with more severe complaints.
- Patients receive sufficient support (eg, CKD-related knowledge and coaching) from the community health care center.

**Organizational**

- There is less avoidable health care use in primary and secondary care.

**Community**

- CKD-related knowledge and treatment are available to community members.
- There is improved collaboration between primary and secondary care in CKD management.

**Public policy**

- The policy of digital health implementation in health care is more concrete.
- There is improved attention to digital health to improve primary care for patients with CKD in public policy.

**Performance objectives (POs; the required actions to accomplish change in behavioral and environmental outcomes) of outcomes.**

**Individual**

- PO 1: patients improved their illness perception.
- PO 2: patients and health care professionals (HCPs) improve their knowledge of chronic kidney disease (CKD) self-management.
- PO 3: patients improve their CKD self-management behavior after a 9-month intervention period.
  PO 3.1: self-integration PO 3.1.1: patients avoid habits that may affect kidney function, such as smoking, drinking, and high-salt food consumption. PO 3.1.2: patients manage food portions and choices in social activities. PO 3.1.3: patients manage food following care providers’ suggestions. PO 3.1.4: patients give up bad habits that are harmful to the kidneys (eg, smoking and drinking alcohol). PO 3.1.5: patients adjust CKD care and lifestyle to fit new situations and maintain the best condition, for instance, diet, physical activity, and medication use. PO 3.1.6: patients manage CKD to stay healthy. PO 3.1.7: patients incorporate CKD management into daily life.
  PO 3.2: problem-solving PO 3.2.1: patients actively seek information about kidney disease and how to better control kidney disease by using multiple resources, such as the internet and books. PO 3.2.2: patients use different ways to clarify questions about treatment plans and solve problems. PO 3.2.3: patients find out reasons for signs and symptoms of health problems, for instance, high blood pressure. PO 3.2.4: patients think over reasons for bad laboratory results, for instance, serum creatinine. PO 3.2.5: patients actively understand the meaning of laboratory data. PO 3.2.6: patients actively understand risk factors of CKD, for instance, high blood pressure, diabetes, and medications.
  PO 3.3: seeking social support PO 3.3.1: patients will share experiences with other patients about how to control kidney disease. PO 3.3.2: sharing helpless and frustrated feelings with other patients, family, or friends PO 3.3.3: patients discuss with family or friends while questioning or worrying about kidney disease and solutions. PO 3.3.4: patients tell family or friends about treatment plans such as diet control and medication use to receive cooperation and support.
  PO 3.4: adherence to recommended regimens PO 3.4.1: patients follow care providers’ suggestions to adjust diet habits, control weight, exercise, and choose food.
- PO 4: patients receive sufficient web-based support and have less face-to-face contact with HCPs.

**Interpersonal**

- PO 5: patients receive sufficient family and peer support during the process of CKD self-management.
- PO 6: patients receive sufficient web support and have less face-to-face contact with HCPs for small complaints. Therefore, HCPs can have sufficient time to focus on patients with more severe complaints.
- PO 7: patients receive sufficient support (eg, CKD-related knowledge) from the community health care center.

**Organizational**

- PO 8: there is less health care use in primary and secondary care.

**Community**

- PO 9: CKD-related knowledge and treatment are available in the community.
- PO 10: there is an improved collaboration between primary and secondary care in CKD management.

**Public policy**

- PO 11: the policy of digital health implementation in health care is more concrete.
- PO 12: there is improved attention to digital health to improve primary care in public policy.

#### Step 3: Selecting Theory-Informed Intervention Methods and Practical Strategies

The selected theoretical methods and practical strategies applied to specific determinants are presented in Multimedia Appendix 3. For instance, the determinant *Beliefs about capabilities* and the PO *Patients improve their CKD self-management behavior after a 9-month intervention period* were linked to the change objective *Patients express confidence in their ability to manage food intake following care providers’ suggestions*. The BCTs that target this change objective include *feedback*, *self-monitoring of the behavior*, and *behavioral practice*. A diary to self-monitor and review progress is included as a practice strategy. Examples of theories mapping to change objectives and translated into practical strategies are shown in Table 1.

**Table 1:** Theoretical methods and practical strategies for change objectives.

Determinant and change objective		PO^a^	Theoretical method	Practical strategy
**Knowledge—patients know**				
	CKD^b^-related knowledge	PO 2	Feedback on behavior Feedback on outcome of behavior Information about patients’ behavior Information about the health consequences of behavior (BCTs^c^)	Lecture Group discussion Program handbook Picture and handout of CKD and self-management knowledge Local community resource handout Workbook
	CKD self-management–related knowledge	PO 2	Feedback on behavior Feedback on outcome of behavior Information about patients’ behavior Information about the health consequences of behavior (BCTs)	Lecture Group discussion Program handbook Picture and handout of CKD and self-management knowledge Local community resource handout Workbook
	What behaviors will negatively affect kidney function and ability to identify the health risk behaviors relevant to them	PO 3.1.1	Feedback on behavior Feedback on outcome of behavior Information about patients’ behavior Information about the health consequences of behavior (BCTs)	Lecture Group discussion Program handbook Picture, handout of CKD and self-management knowledge Local community resources handout Workbook
	Portions and choices of food	PO 3.1.2	Feedback on behavior Feedback on outcome of behavior Information about patients’ behavior Information about the health consequences of behavior (BCTs)	Lecture Group discussion Program handbook Picture, handout of CKD and self-management knowledge Local community resources handout Workbook
	Suggestions for managing food from care providers	PO 3.1.3	Feedback on behavior Feedback on outcome of behavior Information about patients’ behavior Information about the health consequences of behavior (BCTs)	Lecture Group discussion Program handbook Picture, handout of CKD and self-management knowledge Local community resources handout Workbook
	The meaning of laboratory data	PO 3.2.4	Feedback on behavior Feedback on outcome of behavior Information about patients’ behavior Information about the health consequences of behavior (BCTs)	Lecture Group discussion Program handbook Picture, handout of CKD and self-management knowledge Local community resources handout Workbook
	The importance of sharing feelings	PO 3.3.2	Feedback on behavior Feedback on outcome of behavior Information about patients’ behavior Information about the health consequences of behavior (BCTs)	Lecture Group discussion Program handbook Picture, handout of CKD and self-management knowledge Local community resources handout Workbook
	The importance of discussion with family and friends	PO 3.3.3	Feedback on behavior Feedback on outcome of behavior Information about patients’ behavior Information about the health consequences of behavior (BCTs)	Lecture Group discussion Program handbook Picture, handout of CKD and self-management knowledge Local community resources handout Workbook
**Knowledge—HCPs^d^ know**				
	CKD self-management–related knowledge	PO 2	Feedback on behavior Feedback on outcome of behavior Information about patients’ behavior Information about the health consequences of behavior (BCTs)	Lecture Group discussion Program handbook Picture, handout of CKD and self-management knowledge Local community resources handout Workbook

^a^PO: performance objective; the required actions to accomplish change in the behavioral and environmental outcomes.

^b^CKD: chronic kidney disease.

^c^BCT: behavior change technique.

^d^HCP: health care professional.

#### Step 4: Develop a Tailored MD-Based Intervention (Plan)

##### Guiding Principles

Guiding principles are formulated for developing and adapting the MD-based self-management intervention (plan) for the Chinese context. For instance, the tailored MD-based intervention should meet individual patient needs, perceptions, and preferences regarding CKD self-management and focus on the knowledge of, motivation toward, and skills for CKD self-management. Details are summarized in Multimedia Appendix 4.

##### Intervention Description

In China, it is a regular practice that all patients with CKD (stages 1-5 and dialysis) visit a nephrologist in a hospital setting. Patients undergoing a kidney transplant visit a different department, named the Department of Kidney Transplantation. In our needs assessment to prepare for the intervention, we included patients who visited the Department of Nephrology (hence, those in stages 1-5 and undergoing dialysis). As a result, our intervention targets patients with Kidney Disease: Improving Global Outcomes staging of CKD (stages G1-G5 and dialysis) and not patients undergoing kidney transplantation. In addition, as all patients with CKD report a multitude of symptoms and fairly high disease burden, the needs of patients with CKD (regardless of the CKD stage) are basic and similar for self-management interventions. For instance, we found that patients expressed the need for better access to and provision of disease-related knowledge. The level of knowledge of patients with CKD in China is much lower than that of those living in high-income countries. Therefore, in contrast to high-income countries, in China, we need a fairly basic self-management intervention focusing on knowledge, awareness, and basic skills that is suitable for all patients with CKD in stages 1 to 5.

**Figure 2:**
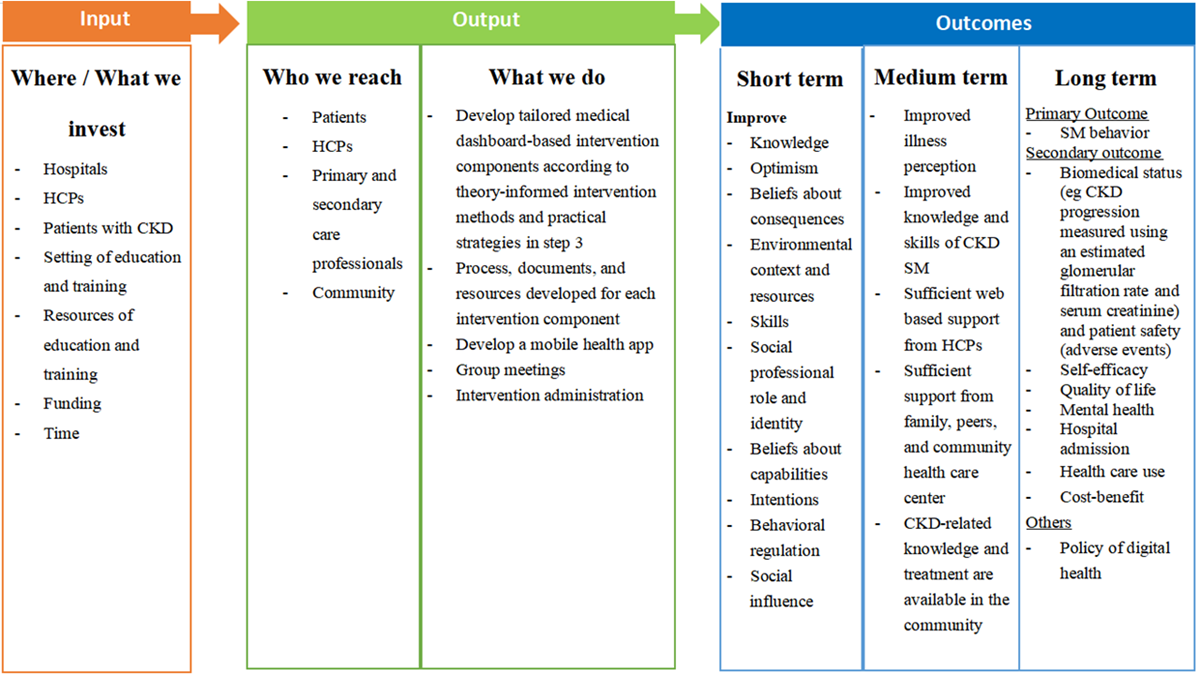
A logic model of a tailored Medical Dashboard–based self-management intervention for patients with chronic kidney disease (CKD). HCP: health care professional; SM: self-management.

A logic model explains how a tailored MD-based self-management intervention (plan) contributes to a chain of results (short-, medium-, and long-term outcomes) that achieve specific program outcomes of our intervention. On the basis of logic model guidance [60], in our program logic model, the short-term outcomes examine changes directly connected to intervention implementation, typically including knowledge, skills, or attitudes that contribute to medium-term outcomes. These are the prerequisite changes expected as a result of the intervention implementation. Medium-term outcomes are specific, measurable changes in things such as certain behaviors, decision-making practices, and community resources, acting as a bridge between short- and long-term outcomes. Long-term outcomes are ultimate changes or impacts, typically including improved health behavior and health conditions, increased capacity, and changes in programmatic reach. The logic model components were developed based on evidence from all previous steps, and a consensus was reached during intervention monitoring group meetings (Figure 2). Details on logic model definitions and examples are shown in Multimedia Appendix 5. On the basis of previous literature, the main concept of a logic model is a cascading effect of the outcomes; long-term outcomes are built (and depend) on medium-term outcomes, which are, in turn, built on short-term outcomes. For outcome evaluation, we selected an intervention duration of 9 months to evaluate long-term outcomes as previous literature provides evidence that this intervention duration is sufficient to demonstrate the impact on several self-management outcome indicators [61,62].

A Chinese MD-based self-management intervention (plan) was built based on the Dutch MD self-management intervention (components; refer to the Dutch MD intervention in Multimedia Appendix 6). Examples of intervention adaptations and their rationale are described in Table 2 (details are provided in Multimedia Appendix 7). Surface-level adaptations included customizing intervention content, messages, and approaches to the local patient population’s cultural characteristics. Our main surface adaptations included (1) providing CKD information translated into Chinese, (2) providing video or voice call options on the dashboard for interactions with HCPs, (3) extending the intervention delivery medium to a mobile phone app combined with a wireless tracker to collect measurements automatically, and (4) a user interface platform for data visualization and progress review.

**Table 2:** Examples of intervention adaptations and priorities of eHealth features.

Original MD^a^ intervention component	Rationale of adaptation	Description of the adapted intervention component
Education	Educational materials and instructions for eHealth use need to be available in Chinese and include relatable, engaging, and relevant formats to Chinese patients. Considering some vulnerable groups’ low health literacy level, face-to-face personal assistance and coaching methods should be used. The need for tailored education and training on the core concepts and possible advantages of self-management interventions was highlighted.	Patients received self-management education, a kidney-friendly cookbook, and an eHealth handbook with instructions on eHealth use. Blended learning that combines e-learning and face-to-face methods supports self-management education development for patients and HCPsb, such as lectures, group discussions, and community resource handouts on self-management and eHealth use. Educational activities of eHealth simulation exercises and real-life practice are provided. Dialysis section: education is also provided specifically on dialysis, such as dialysis introduction, uses, how it works, types, effectiveness, side effects, and impact on a regular routine.
Self-monitoring	The computer-based version of MD was challenging to use as it differed from patients’ previous experience with eHealth technology (ie, smartphone). China has 1.3 billion mobile phone users (penetration rate of 90%), so it is necessary to extend the intervention delivery medium to a mobile phone app combined with a wireless tracker to collect measurements automatically. Patients expressed the need to contact HCPs when their parameters are bad.	Patients are instructed to take health measurements at home (eg, blood pressure) and enter the results via the secure “patient self-care” mobile phone app. Dialysis section: patients with dialysis are instructed to measure or enter the tailored dialysis-related measurements, such as kt/V, serum creatinine, hemoglobin, parathyroid hormone, serum albumin, and serum ferritin. The measurements entered via this patient self-care app are linked in real time to the HCPs’ interface in this app, and the progress is recorded. Alerts are sent to HCPs when patient-entered data are bad and (web-based) communication is provided.
Information support	The web-based disease-related information, tips, suggestions, and adaptations of educational self-management materials need to be available in Chinese. Face-to-face methods for personal assistance and coaching are needed. Smartphone-based informational support is needed. A need for a web-based information platform established by the government or hospital was highlighted.	Patients received chatbot-based support, including web-based disease-related information, tips, and suggestions focusing not only on medical knowledge but also on obtaining and sustaining social support, refusal skills, medication adherence strategies, physical exercise, healthy eating, smoking cessation, and reduced alcohol intake. Lectures, group teaching, and community resource handouts are provided. Dialysis section: the dialysis care team, including the nephrologist (kidney physician), nephrology nurse, and renal dietitian, is online for contact to answer patients’ questions.

^a^MD: Medical Dashboard.

^b^HCP: health care professional.

Personalized features were added, such as visual aids, pictograms, and customized videos (eg, videos covering tailored information about CKD and its treatment, such as a video on fluid restrictions and dialysis procedure for patients with end-stage renal disease or success stories of self-management for newly diagnosed patients with CKD). In addition, the core self-management intervention components that underlie its effectiveness, such as self-monitoring, were not changed. On the basis of the technology functionality framework, priorities of eHealth features of Chinese MD and possible app functionalities were also depicted (Table 3).

**Table 3:** Intervention components and priorities of eHealth features.

Intervention component	Priorities of eHealth features	Possible app functionalities
Education	Improve patients’ access to “easily understandable information” eHealth resources are trustworthy and safe Provide self-management introduction	Education: build a functional module named “Knowledge center” for the patient interface Education: build a functional module named “Knowledge center” for the HCP^a^ interface
Motivational interviewing and modeling	Provide role model stories	Education: build a functional module named “Your story” for the patient interface
Self-monitoring	Track the changes in parameters or symptoms of patients Feedback received from the device on recorded behavior or other personal data (eg, nutrition analysis for daily diet) from eHealth and HCPs Alert sent to patients and HCPs when patient-entered data are bad HCPs’ access to patients’ self-monitored health indicators Wireless tracker in a mobile app to automatically collect measurements A user interface platform in a mobile app to visualize data and review progress Providing video or voice call options on the app to support interactions with HCPs	Record, display, and alert: build a functional module named “My self-monitoring” for the patient interface Communicate: build a functional module named “My medical resources” for the patient interface Display and alert: build a functional module named “My patient” for the HCP interface
Combination of home and hospital measurements	A medium for patients to access their health records	Record and display: build a functional module named “My self-monitoring”
(Web-based) Informational support	The information in eHealth should be easily understandable and illustrate practical medical advice with videos or animations Information in eHealth should be trustworthy and safe Supporting patient communication with HCPs outside of clinical visits Enabling more individualized informational support Animations or videos without medical terminology A reliable, trustworthy, and literacy-appropriate information source	Education: build a functional module named “Knowledge center” Communicate: build a functional module named “My medical resources” Display and alert: build a functional module named “My patient” for the HCP interface

^a^HCP: health care professional.

Furthermore, the intervention monitoring group intended to develop a mobile app to enhance self-management in patients with CKD based on patients’ and HCPs’ needs. The final adapted mobile app contained different modules, as illustrated in Table 4. Considering the low health literacy level in some vulnerable groups, strategies such as keyword searching and animations or videos without medical terminology were used to simplify the content, reduce the complexity, and improve the accessibility and usability of the app content.

**Figure 3:**
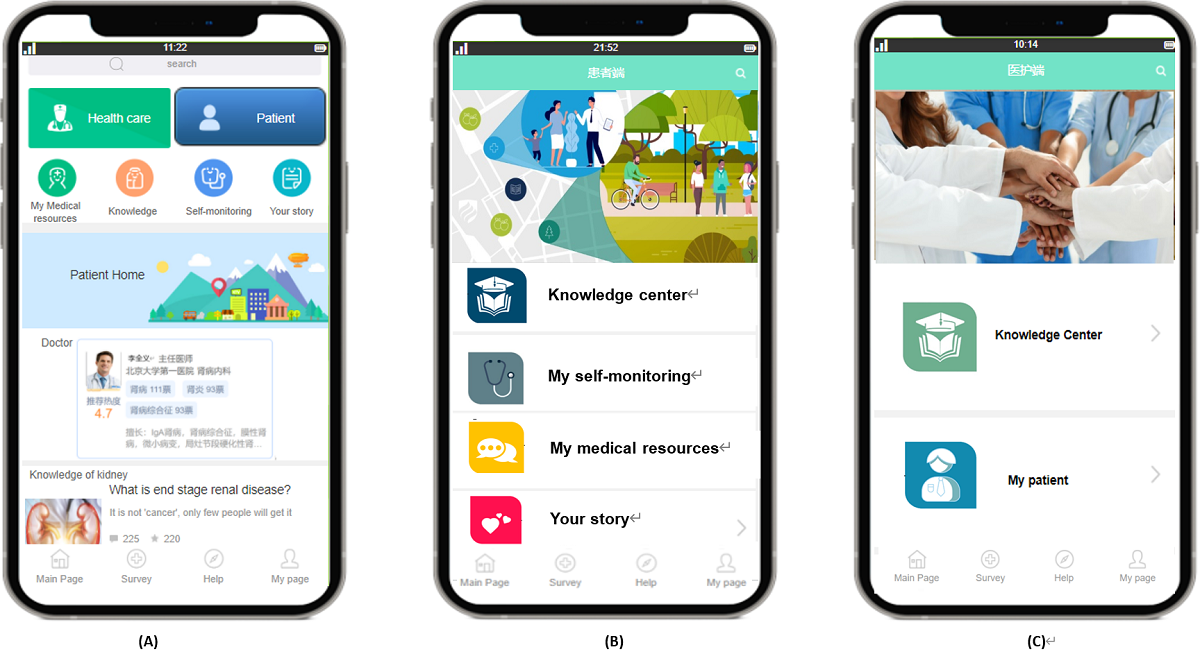
Main page and patient and health care provider user interface of the adapted mobile app for chronic kidney disease self-management. (A) Main page; (B) patient user interface; and (C) health care provider user interface.

**Figure 4:**
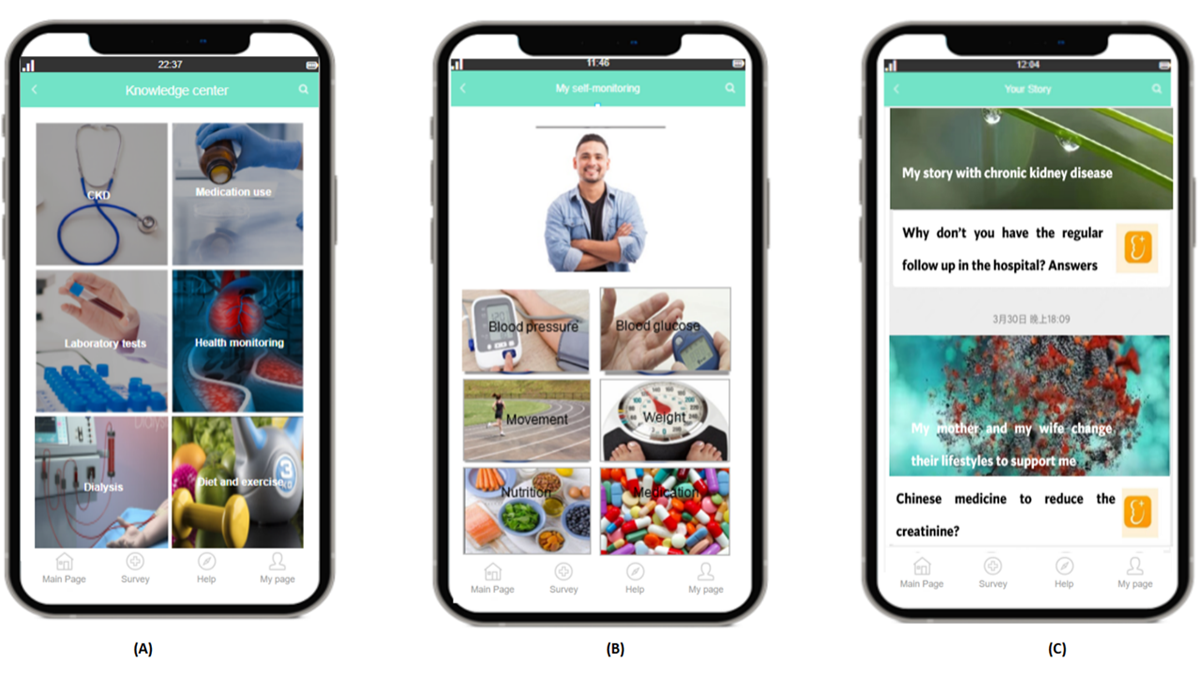
Modules of the adapted mobile app for chronic kidney disease self-management. (A) “Knowledge center” in the patient user interface; (B) “My self-monitoring” in the patient user interface; and (C) “Your story” in the patient user interface.

Low-fidelity prototypes were used to develop the flow, content, and design styles of the app, with examples shown in Figures 3 and 4.

**Table 4:** A summary of modules of the adapted mobile app for chronic kidney disease (CKD) self-management.

Module			Topic		Content
**Patient**					
	1	My page		Introductory section: 60-minute in-person or group session, introduction and intervention overview, practical exercise example, and supports with downloading and using the intervention. Personal information such as disease-related information and user records of app use. My favorite: personalized and accessible preferred content.	
	2	Knowledge center		An intelligent question-answering system—chatbot-based information support system is developed through the knowledge graph. Patients can enter questions in the knowledge center module of the patient app to receive timely and automatic tailored responses. Automatic knowledge delivery: based on patient disease stage, related knowledge is regularly delivered through the knowledge center module to the patient. Browse information: patients can search keywords to browse medical knowledge but also how to attain and sustain self-management behavior such as physical exercise.	
	3	My self-monitoring		Home measurement: patients are instructed to take health measurements at home (eg, blood pressure) using a wireless tracker and enter the results via this module. Hospital measurement: patients access their hospital records. Patient measurements at home and those performed during hospital visits are visualized jointly and reviewed. Alert sent to patients and HCPs^a^ when patient-entered data are bad	
	4	My medical resources		Feedback received from the device on recorded behavior or other personal data (eg, nutrition analysis for daily diet) from HCPs Providing video or voice call options on the app to support interactions with HCPs	
	5	Your story		Serves as an interaction section to facilitate communication among patients, peers, and HCPs to understand patients’ emotional status and apply feasible psychological self-management strategies. For instance, patients can post and browse posts or communicate about experiences with disease management. Role-modeling stories	
**HCP**					
	1	My page		Introductory section, personal information, and “My favorite”: same as the patient user interface	
	2	Knowledge center		Provides self-management introduction and strategies to help patients improve their self-management.	
	3	My patient		Easy access to patients’ self-monitored health indicators and tracking of the changes in parameters or symptoms of patients Feedback on patients’ recorded behavior or other personal data (eg, nutrition analysis for daily diet) Providing video or voice call options on the app to support interactions with patients Alert sent to HCPs when patient-entered data are bad Visualizing data and reviewing progress of patients’ measurements An internet-based section to see the “Your story” module, give feedback on patients’ experience with CKD self-management, and communicate with other HCPs	
**Others**					
	1	User feedback		This section asks patients and HCPs to evaluate the module from the perspective of accessing frequency, subjective experience, and suggestions, helping improve the module further.	
	2	Help		In the “Help” tab, a platform user guideline was designed, illustrating the function and content of each module and the operating procedure.	
	3	Survey		In the “Survey” tab, we set out survey questionnaires, including demographic information, quality of life, and self-management self-efficacy questionnaires.	
	4	Content management system		The content management system is managed by a multidisciplinary team mainly composed of the researchers and network engineers. The tasks include solving app-related problems, data backup, and data statistics. The information that users upload through the “Your story” section and the questions raised through the “Knowledge center” are reviewed and processed to the platform. In addition, the knowledge is delivered to patients.	

^a^HCP: health care professional.

##### Step 5: Develop an Adoption and Implementation Plan

The CFIR-ERIC Matching Tool [59] generates a list of potential strategies to address determinants. An output table (condensed to include highly endorsed strategies) lists the CFIR determinants across the top of the table, with CFIR-ERIC implementation strategies in the first column. Strategies are sorted by cumulative level of endorsement. “Cumulative percent” indicates the strength of endorsement for that strategy across all CFIR determinants (Multimedia Appendix 8). For example, “Identify and prepare champions” has the highest cumulative endorsement (407%) for this strategy to address all determinants. This strategy is color coded in green for all determinants, indicating that most respondents endorsed this strategy for those determinants. The yellow color coded cells indicate that at least 20% of respondents endorsed that strategy to address each indicated barrier. After discussion with the intervention monitoring group, the finalized implementation strategies are detailed in Textbox 4. For instance, to optimize the complexity and compatibility with HCPs’ workflows, ERIC implementation strategies including “develop a formal implementation blueprint,” “conduct local consensus discussions,” “organize clinician implementation team meetings,” and “provide clinical supervision” can be used. These implementation strategies can improve the implementation practice by assessing intervention-workflow compatibility (eg, staff working patterns and practice management) before and during the development and implementation of eHealth interventions and replacing existing care elements instead of adding elements to care.

**A summary of implementation strategies of the Medical Dashboard–based intervention (plan) to improve self-management of patients with chronic kidney disease.**

**Tailored implementation outcomes**

- Proper training and tailored tutorials are needed to guide eHealth implementation to optimize knowledge and skills and promote intervention uptake.
- Improving patients’ eHealth literacy.
- The intervention is easy to use by patients and fits well with and supports health care professionals’ clinical workflows.
- The necessary technology to implement the intervention is available and usable.
- eHealth functionalities must be simple and easy to use, and eHealth navigation must be clear.
- Promoting good relations with family members and the patient’s community.

**Expert Recommendations for Implementing Change strategies**

- Identify and prepare champions
- Conduct educational meetings
- Conduct local consensus discussions
- Develop a formal implementation blueprint
- Develop educational materials
- Conduct ongoing training
- Identify early adopters
- Conduct educational outreach visits
- Provide local technical assistance
- Organize clinician implementation team meetings
- Distribute educational materials
- Involve patients, consumers and family members
- Obtain and use patient or consumer and family member feedback
- Develop and implement tools for quality monitoring
- Make training dynamic
- Funding for clinical innovation
- Recruit, designate, and train leadership
- Audit and provide feedback
- Change physical structure and equipment
- Develop academic partnerships
- Involve executive boards
- Provide clinical supervision
- Develop and organize quality monitoring systems
- Intervene with patients or consumers to enhance uptake and adherence

## Discussion

### Principal Findings

This study used the IM approach to develop a tailored eHealth self-management intervention for patients with CKD in China based on the Dutch MD intervention. The adapted core intervention components comprise education, motivational interviewing, modeling, self-monitoring, combined home and hospital measurements, blended information support, and coaching. The tailored mobile health (mHealth) self-management intervention has potential for use by patients and HCPs in China, improving patient self-management and implementation success. Our study contributes to implementation research on eHealth self-management interventions in CKD care, addressing patient and HCP needs and priorities. It also provides insights into potential barriers and facilitators specific to an eHealth CKD self-management intervention in the Chinese context, which may be relevant for countries with similar contextual characteristics.

### Comparison With Prior Work

#### Identification of Key Barriers and Facilitators

Our findings on key barriers and facilitators of eHealth interventions and self-management in the Chinese context align with those of similar studies in Western contexts. A lack of knowledge among patients was a barrier to CKD self-management, consistent with previous literature [63]. In addition, our finding that patients were generally convinced of the good quality and advantage of eHealth interventions has been corroborated by other research [64]. These similarities suggest that certain challenges such as clinical compatibility persist across different regions and time in the rapidly evolving field of eHealth.

However, our findings also revealed differences from studies in Western settings [21], namely, “Cultural context” (ie, paternalistic patient-HCPs relationship) and “Needs and resources” (ie, patients’ and HCPs’ specific needs in the Chinese context and infrastructure of [primary] health care) in the domain “Outer setting.” For instance, patient autonomy is a core principle of patient-physician interaction in Western cultures [65,66]. However, the appreciated paternalistic relationship in our study can be valuable and even essential to improving health outcomes and treatment adherence in some cultural contexts [67,68]; for instance, if patients prefer and express needs for a paternalistic approach over autonomy [67,68], HCPs can provide guidance on self-management, raising awareness of its importance and potential benefits. This can optimize the effectiveness of the self-management intervention.

Furthermore, improved primary health care infrastructure is needed to support CKD self-management in China. In China, the care for patients with CKD relies heavily on nephrology departments, and the lack of a strong primary care system in rural areas poses a significant challenge due to the distance to health care facilities. Therefore, eHealth interaction plays a crucial role in enabling access to care. These factors can be used to accelerate the implementation of eHealth CKD self-management interventions in countries with similar contextual characteristics to those of China.

#### Theoretical Foundation

Previous studies recommend that a solid theoretical foundation is necessary to plan, design, evaluate, and implement eHealth self-management interventions [69]. However, our review found that only a few eHealth CKD self-management interventions reported the use of a specific theory [21], thereby limiting the understanding of mechanisms of action and hindering replication of eHealth intervention implementation. Our study highlighted the strengths of the theories used.

The IM method guides the development and implementation of interventions by making evidence-based decisions and matching methods to change objectives. It provides an overview of these methods [36]. In addition, the IM-based design involves analyzing relevant constructs in theory, mapping them to stakeholders’ needs, and conceptualizing intervention components. These components are then converted into different functionalities and incorporated into various function modules in the mobile app.

Furthermore, to translate interventions into different contexts (eg, health care system and population), it is crucial to adapt and align these interventions with context characteristics. The HBM and TPB are 2 highly cited social psychological theories focusing on individuals’ perceptions, attitudes, needs, and sociocultural context [70,71]. In step 1, we conducted a needs assessment based on elements mainly from the HBM and TPB to improve intervention alignment with the target population’s needs and priorities. In step 2, we used the CFIR to identify key factors influencing the implementation of eHealth CKD self-management interventions. The CFIR provides a pragmatic structure for identifying potential intervention components and implementation strategies in health systems at multiple levels. In addition, it helped address the black-box problem common in many adaptation models, in which the procedures used to carry out the adaptation process with stakeholders are often vague and unspecified and, thus, difficult to replicate in the new target population and local setting.

### Stakeholder Involvement in Intervention Development and Adaptation

Researchers and intervention developers integrated the perceptions, attitudes, and needs of patients and HCPs to adapt a tailored eHealth self-management intervention for patients with CKD in China. To our knowledge, our study is the first to consider patients’ and HCPs’ needs and preferences in designing and implementing an eHealth intervention for CKD self-management in China. However, to ensure a successful and sustainable implementation of eHealth interventions within a medical organization, it is important to follow patient-oriented principles and have active patient involvement in the research process [72]. In addition, using cocreation methods and engaging patient stakeholders as research partners should be done throughout intervention development and adaptation [73-75].

### MD Self-Management Intervention Adaptation in China

To ensure the effectiveness of the Dutch MD–based self-management intervention, we did not change the core self-management intervention components of MD that underlie its effectiveness, such as provision of information support. In addition, we made surface-level cultural adaptations to customize the intervention content, messages, and approaches to the local patient population, enhancing its appeal and feasibility. Our adaptations focused on the following areas.

First, self-management is often misunderstood as adherence to medical treatment by patients with CKD and HCPs in China [39]. To address this, we included specific self-management training for patients and HCPs in the adapted intervention. Second, Chinese society is more collective compared to most Western countries, emphasizing the importance of good relationships with family and the community. Therefore, the adapted intervention provides information on locally available resources, such as newsletters or role-modeling stories of CKD self-management offered by the community or patient associations.

Third, we adapted the intervention delivery medium to a mobile phone app. mHealth using technologies such as trusted medical knowledge resources can improve telecommunication and behavior monitoring and offer timely and accessible primary health care on a wide scale. This could reduce the burden of CKD, particularly in China, which has numerous internet and mobile phone users. This finding is reflected in previous literature on the development of a user-centered eHealth app in China [76].

Furthermore, we improved accessibility of self-management support for most patients, including vulnerable groups such as people with a lower educational level and eHealth illiteracy and of older age. While eHealth has been proven effective in improving health care locally, regionally, and worldwide [30], some vulnerable populations may be disadvantaged by eHealth; for instance, people who are unemployed or with low education benefit less from web-based interventions [77]. As face-to-face contact remains necessary to optimize medical care, adherence, and treatment outcomes, a “blended or hybrid care model” [78] is applied when necessary, combining eHealth with face-to-face support to provide patients with in-person training on the use of the mHealth app in addition to on-demand web-based help, phone support, or support from family and friends.

### Transferability and Recommendations

Our research extends the translation of potentially effective mHealth interventions to different local contexts, increasing their generalizability. The development and adaptation process encompassed high user involvement, stakeholder input, and robust theories from established eHealth self-management interventions, enhancing the intervention’s potential effectiveness. In addition, the methodological approach and findings of our study are relevant to the Chinese context as well as other countries with similar contextual characteristics such as the lack of a strong primary care system, long traveling distances to health care facilities, a limited understanding and knowledge of CKD (self-management), a more prominent paternalistic patient-HCP relationship, and wide-scale use of mobile phone apps.

The lessons learned from our study can guide future research on intervention adaptation. First, instead of a “one-size-fits-all” approach, researchers and eHealth intervention developers should be aware of context-specific factors in the local settings. Second, this study serves as an example of preparation for intervention adaptation by leveraging the experiences of clinicians and multidisciplinary teams to produce the initial prototype app.

### Strengths and Limitations

Our study has several strengths. To our knowledge, it is the first to use the IM approach to develop and adapt an eHealth intervention for CKD self-management in China. The IM approach incorporates theory and evidence, providing guidance for local adaptation. Second, we provide an overview of barriers and facilitators specific to eHealth self-management interventions in the Chinese context. Third, our research team is multidisciplinary, including academic general practitioners, a nephrologist, health services researchers, nurses, and a behavioral scientist. The involvement of a multidisciplinary intervention monitoring group ensured the intervention’s feasibility and alignment with existing processes.

Nevertheless, there are also limitations. First, we did not include primary care HCPs in the qualitative interviews. In rural China, where a robust primary care system is lacking, the responsibility for CKD care primarily lies with HCPs in nephrology departments at city hospitals. As a result, patients with CKD have limited or no contact with their primary care providers. Second, we performed a needs assessment to identify perceptions, attitudes, and needs of patients and HCPs regarding eHealth self-management interventions for CKD. The participation of those that matter is essential for IM and greatly determines the success of the intervention in practice. However, due to limited time and resources, we unfortunately were not able to include patients and caregivers in the intervention monitoring group. For future research, we will include them in the monitoring group, such as using the “member check” method to explore patients’ and caregivers’ views on the intervention plan and provide an in-depth understanding of its usability, functionality, and acceptability. Third, the intervention development study is based exclusively on formative research methods, and a pilot test is currently underway to optimize the MD-based self-management intervention in preparation for a full-scale randomized controlled trial.

### Conclusions

eHealth interventions such as the Dutch MD self-management intervention could be widely used to improve the health of people with CKD. To increase the reach of these interventions in the public health system, especially in low- and middle-income countries, adaptations to the local context are needed. This paper details the development and adaptation process of the Dutch MD–based CKD self-management intervention to the Chinese setting. By systematically applying an IM approach collaborating with experts and stakeholders, an mHealth-based CKD self-management intervention was developed that provides a good fit with patients’ needs and priorities and is easy to use and well integrated into HCPs’ workflows. To translate the implementation of effective eHealth self-management interventions for patients in local settings, we recommend that future researchers and intervention developers explore the intersection of factors that influence the use of eHealth (eg, previous experience of eHealth use and preferences) to discern tailored intervention adaptation. In addition, the iterative Plan-Do-Study-Act cycle is needed for continuous intervention improvement in daily practice that aims to identify barriers and needed changes in implementation and outcome efforts.

## Appendix

Multimedia Appendix 1 Key lessons learned from the needs assessment.

Multimedia Appendix 2 Performance objectives, determinants, and change objectives of outcomes.

Multimedia Appendix 3 Theoretical methods and practical strategies for change objectives.

Multimedia Appendix 4 Guiding principles for a tailored Medical Dashboard–based intervention (plan) to improve self-management in patients with chronic kidney disease.

Multimedia Appendix 5 Logic model definitions and examples for a tailored Medical Dashboard–based self-management intervention for patients with chronic kidney disease.

Multimedia Appendix 6 Core intervention components and functionalities of the medical dashboard.

Multimedia Appendix 7 A summary of intervention adaptations and priorities of eHealth features.

Multimedia Appendix 8 Consolidated Framework for Implementation Research (CFIR)–Expert Recommendations for Implementing Change Matching Tool for strategies across all CFIR determinants.

## Abbreviations

BCT: behavior change technique
CFIR: Consolidated Framework for Implementation Research
CKD: chronic kidney disease
ERIC: Expert Recommendations for Implementing Change
HBM: Health Belief Model
HCP: health care professional
IM: intervention mapping
MD: Medical Dashboard
mHealth: mobile health
PO: performance objective
SETTING: Setting Exploration Treasure Trail to Inform Implementation Strategies
TPB: Theory of Planned Behavior

## Data Availability

The data sets generated during and analyzed during this study are available from the corresponding author on reasonable request.

## References

[1] Webster AC, Nagler EV, Morton RL, Masson P (2017). Chronic kidney disease. Lancet.

[2] George C, Mogueo A, Okpechi I, Echouffo-Tcheugui JB, Kengne AP (2017). Chronic kidney disease in low-income to middle-income countries: the case for increased screening. BMJ Glob Health.

[3] Carney EF (2020). The impact of chronic kidney disease on global health. Nat Rev Nephrol.

[4] Chin HJ, Song YR, Lee JJ, Lee SB, Kim KW, Na KY, Kim S, Chae D (2008). Moderately decreased renal function negatively affects the health-related quality of life among the elderly Korean population: a population-based study. Nephrol Dial Transplant.

[5] Etgen T, Chonchol M, Förstl H, Sander D (2012). Chronic kidney disease and cognitive impairment: a systematic review and meta-analysis. Am J Nephrol.

[6] Chen SH, Tsai YF, Sun CY, Wu IW, Lee CC, Wu MS (2011). The impact of self-management support on the progression of chronic kidney disease--a prospective randomized controlled trial. Nephrol Dial Transplant.

[7] Mills KT, Xu Y, Zhang W, Bundy JD, Chen CS, Kelly TN, Chen J, He J (2015). A systematic analysis of worldwide population-based data on the global burden of chronic kidney disease in 2010. Kidney Int.

[8] GBD Chronic Kidney Disease Collaboration (2020). Global, regional, and national burden of chronic kidney disease, 1990-2017: a systematic analysis for the Global Burden of Disease Study 2017. Lancet.

[9] Allegrante JP, Wells MT, Peterson JC (2019). Interventions to support behavioral self-management of chronic diseases: a pragmatic randomized controlled trial. Annu Rev Public Health.

[10] Choi ES, Lee J (2012). Effects of a face-to-face self-management program on knowledge, self-care practice and kidney function in patients with chronic kidney disease before the renal replacement therapy. J Korean Acad Nurs.

[11] Peng S, He J, Huang J, Lun L, Zeng J, Zeng S, Zhang L, Liu X, Wu Y (2019). Self-management interventions for chronic kidney disease: a systematic review and meta-analysis. BMC Nephrol.

[12] Zimbudzi E, Lo C, Misso ML, Ranasinha S, Kerr PG, Teede HJ, Zoungas S (2018). Effectiveness of self-management support interventions for people with comorbid diabetes and chronic kidney disease: a systematic review and meta-analysis. Syst Rev.

[13] Lin MY, Liu MF, Hsu LF, Tsai PS (2017). Effects of self-management on chronic kidney disease: a meta-analysis. Int J Nurs Stud.

[14] Lee MC, Wu SV, Hsieh NC, Tsai JM (2016). Self-management programs on eGFR, depression, and quality of life among patients with chronic kidney disease: a meta-analysis. Asian Nurs Res (Korean Soc Nurs Sci).

[15] McManus RJ, Mant J, Haque MS, Bray EP, Bryan S, Greenfield SM, Jones MI, Jowett S, Little P, Penaloza C, Schwartz C, Shackleford H, Shovelton C, Varghese J, Williams B, Hobbs FR, Gooding T, Morrey I, Fisher C, Buckley D (2014). Effect of self-monitoring and medication self-titration on systolic blood pressure in hypertensive patients at high risk of cardiovascular disease: the TASMIN-SR randomized clinical trial. JAMA.

[16] Lee YL, Cui YY, Tu MH, Chen YC, Chang P (2018). Mobile health to maintain continuity of patient-centered care for chronic kidney disease: content analysis of apps. JMIR Mhealth Uhealth.

[17] Fakih El Khoury C, Crutzen R, Schols JM, Halfens RJ, Karavetian M (2020). A dietary mobile app for patients undergoing hemodialysis: prospective pilot study to improve dietary intakes. J Med Internet Res.

[18] Eaton C, Comer M, Pruette C, Psoter K, Riekert K (2020). Text messaging adherence intervention for adolescents and young adults with chronic kidney disease: pilot randomized controlled trial and stakeholder interviews. J Med Internet Res.

[19] Ong SW, Jassal SV, Miller JA, Porter EC, Cafazzo JA, Seto E, Thorpe KE, Logan AG (2016). Integrating a smartphone-based self-management system into usual care of advanced CKD. Clin J Am Soc Nephrol.

[20] Reese PP, Bloom RD, Trofe-Clark J, Mussell A, Leidy D, Levsky S, Zhu J, Yang L, Wang W, Troxel A, Feldman HI, Volpp K (2017). Automated reminders and physician notification to promote immunosuppression adherence among kidney transplant recipients: a randomized trial. Am J Kidney Dis.

[21] Shen H, van der Kleij RM, van der Boog PJ, Chang X, Chavannes NH (2019). Electronic health self-management interventions for patients with chronic kidney disease: systematic review of quantitative and qualitative evidence. J Med Internet Res.

[22] Blakeman T, Blickem C, Kennedy A, Reeves D, Bower P, Gaffney H, Gardner C, Lee V, Jariwala P, Dawson S, Mossabir R, Brooks H, Richardson G, Spackman E, Vassilev I, Chew-Graham C, Rogers A (2014). Effect of information and telephone-guided access to community support for people with chronic kidney disease: randomised controlled trial. PLoS One.

[23] Meuleman Y, Hoekstra T, Dekker FW, Navis G, Vogt L, van der Boog PJ, Bos WJ, van Montfrans GA, van Dijk S (2017). Sodium restriction in patients with CKD: a randomized controlled trial of self-management support. Am J Kidney Dis.

[24] van Lint CL, van der Boog PJ, Wang W, Brinkman WP, Rövekamp TJ, Neerincx MA, Rabelink TJ, van Dijk S (2015). Patient experiences with self-monitoring renal function after renal transplantation: results from a single-center prospective pilot study. Patient Prefer Adherence.

[25] Humalda JK, Klaassen G, de Vries H, Meuleman Y, Verschuur LC, Straathof EJ, Laverman GD, Bos WJ, van der Boog PJ, Vermeulen KM, Blanson Henkemans OA, Otten W, de Borst MH, van Dijk S, Navis GJ (2020). A self-management approach for dietary sodium restriction in patients with CKD: a randomized controlled trial. Am J Kidney Dis.

[26] Kong G, Wang J, Lin H, Bao B, Friedman CP, Zhang L (2023). Transforming health care through a learning health system approach in the digital era: chronic kidney disease management in China. Health Data Sci.

[27] Han Y, Lie RK, Guo R (2020). The internet hospital as a telehealth model in China: systematic search and content analysis. J Med Internet Res.

[28] Tan X, Wu Q, Shao H (2018). Global commitments and China's endeavors to promote health and achieve sustainable development goals. J Health Popul Nutr.

[29] Zhang L, Zhao MH, Zuo L, Wang Y, Yu F, Zhang H, Wang H (2020). China Kidney Disease Network (CK-NET) 2016 annual data report. Kidney Int Suppl (2011).

[30] Xie X, Zhou W, Lin L, Fan S, Lin F, Wang L, Guo T, Ma C, Zhang J, He Y, Chen Y (2017). Internet hospitals in China: cross-sectional survey. J Med Internet Res.

[31] Shen H, van der Kleij R, van der Boog PJ, Wang W, Song X, Li Z, Brakema E, Lou X, Chavannes N (2022). Digital tools/eHealth to support CKD self-management: a qualitative study of perceptions, attitudes and needs of patients and health care professionals in China. Int J Med Inform.

[32] Sadler E, Wolfe CD, McKevitt C (2014). Lay and health care professional understandings of self-management: a systematic review and narrative synthesis. SAGE Open Med.

[33] Brakema EA, van der Kleij RM, Poot CC, Chavannes NH, Tsiligianni I, Walusimbi S, An PL, Sooronbaev T, Numans ME, Crone MR, Reis RR (2021). A systematic approach to context-mapping to prepare for health interventions: development and validation of the SETTING-tool in four countries. BMJ Glob Health.

[34] Bauer MS, Kirchner J (2020). Implementation science: what is it and why should I care?. Psychiatry Res.

[35] Versluis A, van Luenen S, Meijer E, Honkoop PJ, Pinnock H, Mohr DC, Neves AL, Chavannes NH, van der Kleij RM (2020). SERIES: eHealth in primary care. Part 4: addressing the challenges of implementation. Eur J Gen Pract.

[36] Bartholomew LK, Kok GS, Gottlieb NH, Kok G (2006). Planning Health Promotion Programs: An Intervention Mapping Approach. 2nd edition.

[37] Geense WW, van Gaal BG, Knoll JL, Maas NM, Kok G, Cornelissen EA, Nijhuis-van der Sanden MW (2018). Effect and process evaluation of e-powered parents, a web-based support program for parents of children with a chronic kidney disease: feasibility randomized controlled trial. J Med Internet Res.

[38] Garba RM, Gadanya MA (2017). The role of intervention mapping in designing disease prevention interventions: a systematic review of the literature. PLoS One.

[39] Shen H, van der Kleij RM, van der Boog PJ, Wang W, Song X, Li Z, Lou X, Chavannes N (2021). Patients' and healthcare professionals' beliefs, perceptions and needs towards chronic kidney disease self-management in China: a qualitative study. BMJ Open.

[40] Shen H, van der Kleij R, van der Boog PJ, Song X, Wang W, Zhang T, Li Z, Lou X, Chavannes N (2020). Development and evaluation of an eHealth self-management intervention for patients with chronic kidney disease in China: protocol for a mixed-method hybrid type 2 trial. BMC Nephrol.

[41] Keith RE, Crosson JC, O'Malley AS, Cromp D, Taylor EF (2017). Using the consolidated framework for implementation research (CFIR) to produce actionable findings: a rapid-cycle evaluation approach to improving implementation. Implement Sci.

[42] Damschroder LJ, Aron DC, Keith RE, Kirsh SR, Alexander JA, Lowery JC (2009). Fostering implementation of health services research findings into practice: a consolidated framework for advancing implementation science. Implement Sci.

[43] Alderson H, McGovern R, Copello A, McColl E, Kaner E, Smart D, McArdle P, Lingam R (2021). Implementation factors for the delivery of alcohol and drug interventions to children in care: qualitative findings from the SOLID feasibility trial. Int J Environ Res Public Health.

[44] Gustafsson T, Sundler AJ, Lindberg E, Karlsson P, Söderholm HM (2021). Process evaluation of the ACTION programme: a strategy for implementing person-centred communication in home care. BMC Nurs.

[45] Reale S, Turner RR, Sutton E, Taylor SJ, Bourke L, Morrissey D, Brown J, Rosario DJ, Steed L (2021). Towards implementing exercise into the prostate cancer care pathway: development of a theory and evidence-based intervention to train community-based exercise professionals to support change in patient exercise behaviour (The STAMINA trial). BMC Health Serv Res.

[46] Seckler E, Regauer V, Krüger M, Gabriel A, Hermsdörfer J, Niemietz C, Bauer P, Müller M (2021). Improving mobility and participation of older people with vertigo, dizziness and balance disorders in primary care using a care pathway: feasibility study and process evaluation. BMC Fam Pract.

[47] Tully L, Case L, Arthurs N, Sorensen J, Marcin JP, O'Malley G (2021). Barriers and facilitators for implementing paediatric telemedicine: rapid review of user perspectives. Front Pediatr.

[48] Ciere Y, van der Vaart R, van der Meulen-De Jong AE, Maljaars PW, van Buul AR, Koopmans JG, Snoeck-Stroband J, Chavannes N, Sont J, Evers A (2019). Implementation of an eHealth self-management care path for chronic somatic conditions. Clin eHealth.

[49] McLeroy KR, Bibeau D, Steckler A, Glanz K (1988). An ecological perspective on health promotion programs. Health Educ Q.

[50] Michie S, Johnston M, Francis J, Hardeman W, Eccles M (2008). From theory to intervention: mapping theoretically derived behavioural determinants to behaviour change techniques. Appl Psychol.

[51] Ajzen I (1991). The theory of planned behavior. Organ Behav Hum Decis Process.

[52] Rosenstock IM, Strecher VJ, Becker MH (1988). Social learning theory and the Health Belief model. Health Educ Q.

[53] Michie S, Richardson M, Johnston M, Abraham C, Francis J, Hardeman W, Eccles MP, Cane J, Wood CE (2013). The behavior change technique taxonomy (v1) of 93 hierarchically clustered techniques: building an international consensus for the reporting of behavior change interventions. Ann Behav Med.

[54] Baxter SK, Blank L, Woods HB, Payne N, Rimmer M, Goyder E (2014). Using logic model methods in systematic review synthesis: describing complex pathways in referral management interventions. BMC Med Res Methodol.

[55] Korpershoek YJ, Hermsen S, Schoonhoven L, Schuurmans MJ, Trappenburg JC (2020). User-centered design of a mobile health intervention to enhance exacerbation-related self-management in patients with chronic obstructive pulmonary disease (Copilot): mixed methods study. J Med Internet Res.

[56] Powell AC, Landman AB, Bates DW (2014). In search of a few good apps. JAMA.

[57] Chaet AV, Morshedi B, Wells KJ, Barnes LE, Valdez R (2016). Spanish-language consumer health information technology interventions: a systematic review. J Med Internet Res.

[58] Chatterjee A, Prinz A, Gerdes M, Martinez S, Pahari N, Meena YK (2022). ProHealth eCoach: user-centered design and development of an eCoach app to promote healthy lifestyle with personalized activity recommendations. BMC Health Serv Res.

[59] Shin MH, Montano AL, Adjognon OL, Harvey KL, Solimeo SL, Sullivan JL (2023). Identification of implementation strategies using the CFIR-ERIC matching tool to mitigate barriers in a primary care model for older veterans. Gerontologist.

[60] Knowlton LW, Phillips CC (2012). The Logic Model Guidebook: Better Strategies for Great Results.

[61] Nguyen NT, Douglas C, Bonner A (2019). Effectiveness of self-management programme in people with chronic kidney disease: a pragmatic randomized controlled trial. J Adv Nurs.

[62] Lin CC, Tsai FM, Lin HS, Hwang SJ, Chen HC (2013). Effects of a self-management program on patients with early-stage chronic kidney disease: a pilot study. Appl Nurs Res.

[63] Willis MA, Hein LB, Hu Z, Saran R, Argentina M, Bragg-Gresham J, Krein SL, Gillespie B, Zheng K, Veinot TC (2021). Feeling better on hemodialysis: user-centered design requirements for promoting patient involvement in the prevention of treatment complications. J Am Med Inform Assoc.

[64] Donald M, Beanlands H, Straus S, Ronksley P, Tam-Tham H, Finlay J, MacKay J, Elliott M, Herrington G, Harwood L, Large CA, Large CL, Waldvogel B, Sparkes D, Delgado M, Tong A, Grill A, Novak M, James MT, Brimble KS, Samuel S, Hemmelgarn BR (2019). Identifying needs for self-management interventions for adults with CKD and their caregivers: a qualitative study. Am J Kidney Dis.

[65] Emanuel EJ, Emanuel LL (1992). Four models of the physician-patient relationship. JAMA.

[66] Hellín T (2002). The physician-patient relationship: recent developments and changes. Haemophilia.

[67] Carrard V, Schmid Mast M, Cousin G (2016). Beyond "one size fits all": physician nonverbal adaptability to patients' need for paternalism and its positive consultation outcomes. Health Commun.

[68] Thompson GA, Whiffen LH (2018). Can physicians demonstrate high quality care using paternalistic practices? A case study of paternalism in Latino physician-patient interactions. Qual Health Res.

[69] Bezabih AM, Gerling K, Abebe W, Abeele VV (2021). Behavioral theories and motivational features underlying eHealth interventions for adolescent antiretroviral adherence: systematic review. JMIR Mhealth Uhealth.

[70] Chen H, Li X, Gao J, Liu X, Mao Y, Wang R, Zheng P, Xiao Q, Jia Y, Fu H, Dai J (2021). Health belief model perspective on the control of COVID-19 vaccine hesitancy and the promotion of vaccination in China: web-based cross-sectional study. J Med Internet Res.

[71] Li D, Hu Y, Pfaff H, Wang L, Deng L, Lu C, Xia S, Cheng S, Zhu X, Wu X (2020). Determinants of patients' intention to use the online inquiry services provided by internet hospitals: empirical evidence from China. J Med Internet Res.

[72] Lee EW, McCloud RF, Viswanath K (2022). Designing effective eHealth interventions for underserved groups: five lessons from a decade of eHealth intervention design and deployment. J Med Internet Res.

[73] Harrison JD, Auerbach AD, Anderson W, Fagan M, Carnie M, Hanson C, Banta J, Symczak G, Robinson E, Schnipper J, Wong C, Weiss R (2019). Patient stakeholder engagement in research: a narrative review to describe foundational principles and best practice activities. Health Expect.

[74] Hacker KE, Smith AB (2018). Engaging stakeholders and patient partners. Surg Oncol Clin N Am.

[75] Pérez Jolles M, Willging CE, Stadnick NA, Crable EL, Lengnick-Hall R, Hawkins J, Aarons GA (2022). Understanding implementation research collaborations from a co-creation lens: recommendations for a path forward. Front Health Serv.

[76] Ji X, Hu L, Wang Y, Luo Y, Zhu J, Zhang J, Khan MA, Huang F (2019). "Mobile health" for the management of spondyloarthritis and its application in China. Curr Rheumatol Rep.

[77] Latulippe K, Hamel C, Giroux D (2017). Social health inequalities and eHealth: a literature review with qualitative synthesis of theoretical and empirical studies. J Med Internet Res.

[78] Zelst CV, Noort E, Chavannes N, Veen H, Kasteleyn M (2020). Blended care results in an improved adherence of an eHealth Platform by COPD patients. Eur Respir J.

