# Supplementary material for: Developing a Tailored eHealth Self-Management Intervention for Patients With Chronic Kidney Disease in China: Intervention Mapping Approach
Source: JMIR Form Res. 2024 Jun 13;8:e48605. doi: 10.2196/48605 (PMC11211709; doi:10.2196/48605)
Supplement: Multimedia Appendix 1 [file formative_v8i1e48605_app1.docx]

**Multimedia Appendix 1** **Key lessons learned from the needs assessment**

- Educational efforts are needed to ensure patients and HCPs understand the core concepts and potential benefits of self-management and eHealth before implementation.
- As the majority of patients only mentioned performing disease-specific acts of control, an increase focus should be placed on role management and emotional coping skills.
- eHealth CKD self-management intervention components should be tailored by addressing the existing paternalistic patient–HCP relationship. For instance, the paternalistic guidance on self-management provided by HCPs can help patients become aware of the importance and potential benefits of self-management. As such, an optimal effect of the self-management intervention can be achieved.
- eHealth should be developed and implemented as a medium to provide easy access to sufficient disease-related knowledge and trustworthy (online) educational resources, and also support effective patients–HCP communication.
- To increase the clinical compatibility of eHealth interventions, such interventions should partially replace existing care elements and deliver added value to health care.
- To ensure that the eHealth application is time-saving, eHealth functionalities must be simple and easy to use and eHealth navigation must be clear.
- The design and implementation of eHealth CKD self-management intervention should take vulnerable groups and eHealth illiteracy into account.
- Evidence-based implementation strategies are essential to improve eHealth implementation outcomes and optimize implementation success.
