# Supplementary material for: Developing a Tailored eHealth Self-Management Intervention for Patients With Chronic Kidney Disease in China: Intervention Mapping Approach
Source: JMIR Form Res. 2024 Jun 13;8:e48605. doi: 10.2196/48605 (PMC11211709; doi:10.2196/48605)
Supplement: Multimedia Appendix 2 [file formative_v8i1e48605_app2.docx]

**Multimedia Appendix 2 Performance objectives, determinants, and change objectives of outcomes**

| Performance objective (PO) | Details | Determinant | Change objective(s) |
| --- | --- | --- | --- |
| Individual outcome |  |  |  |
| PO.1. Patients improved their illness perception |  | - Optimism | - Patients feel optimistic about their ability to manage chronic kidney disease |
|  |  | - Beliefs about Consequences | - Patients believe that being responsible for self-management of their disease will improve their outcomes and quality of life |
| PO.2. Patients and HCPs improve their knowledge of CKD SM |  | - Knowledge | - Patients have sufficient knowledge about CKD (self-management) |
|  |  | - Beliefs about Consequences | - Patients and HCPs believe that optimalized CKD SM can improve patients’ quality of life and other health outcomes |
|  |  | - Environmental context and resources | - Educational resources and tailored training of CKD SM are available for patients and HCPs |
| PO.3. Patients improve their CKD self-management behavior after a 9-month intervention period |  |  |  |
| PO.3.1. Self-integration (patients’ adjustment to their life style and implementation of recommended regimens and self-care activities to achieve a balanced life) |  |  |  |
|  | PO.3.1.1. Patients heed habits that may affect kidney function, such as smoking, drinking, high-salt eating habits. | - Knowledge | - Patients have sufficient knowledge about what health risk behaviors will negatively affect kidney function, and can identify the health risk behaviors relevant to them |
|  |  | - Skills | - Patients develop the ability to change their health risk behaviors |
|  |  | - Social/professional role and identity | - Patients take responsibility of the performance of health risk behaviors |
|  |  | - Beliefs about capabilities | - Patients express confidence in their ability to mitigate health risk behaviors |
|  |  | - Beliefs about Consequences | - Patients believe that changing health risk behaviors will be beneficial for their health outcomes |
|  |  | - Intentions | - Patients have the intention to change health risk behaviors |
|  |  | - Behavioural regulation | - Patients can identify and practice mitigation strategies to change their health risk behaviorss |
|  | PO.3.1.2. Patients manage food portions and choices in social activity. | - Knowledge | - Patients have sufficient knowledge about the portions and choices of food |
|  |  | - Skills | - Patients develop the ability to manage food portions and choices |
|  |  | - Social/professional role and identity | - Patients take responsibility of choosing food |
|  |  | - Beliefs about capabilities | - Patients express the confidence in ability to manage food portions and choices. |
|  |  | - Beliefs about Consequences | - Patients believe that manage food portions and choices will be beneficial for their health outcomes |
|  |  | - Environmental context and resources | - Educational resources, tailored training and practical applications are available for patients to manage food portions and choices - Nutritionist provide support for patients |
|  |  | - Behavioural regulation | - A patient identify strategies to manage food portions and choices. |
|  | PO.3.1.3. Patients manage food following to care providers’ suggestion | - Knowledge | - Patients have sufficient knowledge about the suggestions of managing food from care providers |
|  |  | - Skills | - Patients develop the ability to manage food |
|  |  | - Social/professional role and identity | - Patients take responsibility of managing food following care providers’ suggestions |
|  |  | - Beliefs about capabilities | - Patients express the confidence in ability to manage food following care providers’ suggestions |
|  |  | - Beliefs about Consequences | - Patients believe that manage food following care providers’ suggestions will be beneficial for their health outcomes |
|  |  | - Environmental context and resources | - Suggestions of care providers are available and sufficient for patients to manage food. |
|  |  | - Behavioural regulation | - A patient identify strategies to manage food. |
|  | PO.3.1.4. Patients give up bad habits which are harmful to kidney (e.g., smoking, drinking alcohol). | - Knowledge | - Patients have sufficient knowledge about what habits are harmful for kidney |
|  |  | - Skills | - Patients develop ability to give up bad habits. |
|  |  | - Social/professional role and identity | - Patients take responsibility of giving up bad habits |
|  |  | - Beliefs about capabilities | - Patients express the confidence in ability to give up bad habits |
|  |  | - Beliefs about Consequences | - Patients believe that giving up bad habits will be beneficial for their kidney and health outcomes |
|  |  | - Behavioural regulation | - A patient identify strategies to give up bad habits |
|  | PO.3.1.5. Patients adjust CKD care and lifestyle to fit new situations and maintain the best condition, for instance, diet intake, physical activity, and medication use. | - Knowledge | - Patients have sufficient knowledge about what CKD care and lifestyle needs to be adjusted. |
|  |  | - Skills | - Patients develop ability to adjust CKD care and lifestyle. |
|  |  | - Social/professional role and identity | - Patients take responsibility of adjusting CKD care and lifestyle |
|  |  | - Beliefs about capabilities | - Patients express the confidence in ability to adjust CKD care and lifestyle |
|  |  | - Beliefs about Consequences | - Patients believe that adjusting CKD care and lifestyle will be beneficial for their health outcomes |
|  |  | - Environmental context and resources | - Suggestions of adjusting CKD care and lifestyle are available |
|  |  | - Behavioural regulation | - A patient identify strategies to adjust CKD care and lifestyle |
|  | PO.3.1.6. Patients take managing CKD as to stay healthy | - Knowledge | - Patients have sufficient knowledge about that the aim of managing CKD is to stay healthy |
|  |  | - Optimism | - Patients express positive on managing CKD |
|  |  | - Beliefs about Consequences | - Patients believe that managing CKD will be help them stay healthy |
|  |  | - Environmental context and resources | - Educational resources and tailored training of managing CKD are available |
|  | PO.3.1.7. Patients merge CKD management into daily life | - Skills | - Patients develop ability to merge CKD management into daily life. |
|  |  | - Beliefs about Consequences | - Patients believe that merging CKD management into daily life will be beneficial for their health outcomes |
| PO.3.2. Problem-solving (This factor reflects the patient’s ability to seek the resources and actively learn  disease-specific knowledge and skills.) |  |  |  |
|  | PO.3.2.1. Patients actively seek information about kidney disease and how to better control kidney disease by using multiple resources, such as internet and books. | - Skills | - Patients develop ability to seek information about kidney disease. |
|  |  | - Environmental context and resources | - Good-quality educational resources are available for patients to seek information about kidney disease. |
|  | PO.3.2.2. Patients utilize different ways to clarify questions about treatment plans and solve problems | - Skills | - Patients develop ability to clarify questions about treatment plan and solve problems |
|  |  | - Social/professional role and identity | - Patients take responsibility of clarifying questions about treatment plan and solve problems |
|  |  | - Beliefs about Consequences | - Patients believe that clarifying questions about treatment and solve problems will be beneficial for their CKD management |
|  |  | - Environmental context and resources | - Resources are available for patients to clarify questions of treatment plan and solve problems |
|  | PO.3.2.3. Patients find out reasons for signs and symptoms of health problems, for instance, high blood pressure. | - Knowledge | - Patients have sufficient knowledge about the signs and symptoms of health problems |
|  |  | - Skills | - Patients develop ability to find out reasons for signs and symptoms of health problems |
|  |  | - Social/professional role and identity | - Patients take responsibility of finding out reasons for signs and symptoms of health problems |
|  |  | - Beliefs about capabilities | - Patients express the confidence in ability to find out reasons for signs and symptoms of health problems |
|  |  | - Beliefs about Consequences | - Patients believe that finding out reasons for signs and symptoms of health problems will be beneficial for their health outcomes |
|  |  | - Environmental context and resources | - Resources are available for patients to find out reasons for signs and symptoms of health problems. |
|  |  | - Social influence | - Patients received sufficient support from HCPs, families and peers. |
|  | PO.3.2.4. Patients think over reasons about bad laboratory data, for instance serum creatinine | - Knowledge | - Patients have sufficient knowledge about the meaning of laboratory data |
|  |  | - Skills | - Patients develop ability to think over reasons about bad laboratory data |
|  |  | - Social/professional role and identity | - Patients take responsibility of thinking over reasons about bad laboratory data |
|  |  | - Beliefs about capabilities | - Patients express the confidence in ability to think over reasons about bad laboratory data |
|  |  | - Beliefs about Consequences | - Patients believe that thinking over reasons about bad laboratory data will be beneficial for their health outcomes |
|  |  | - Environmental context and resources | - Resources are available for patients to find information of laboratory data |
|  |  | - Social influences | - Patients received sufficient support from HCPs, families and peers. |
|  | PO.3.2.5. Patients actively understand the meaning of laboratory data | - Knowledge | - Patients have sufficient knowledge about the meaning of laboratory data |
|  |  | - Social/professional role and identity | - Patients take responsibility of understanding the meaning of laboratory data |
|  |  | - Beliefs about capabilities | - Patients express the confidence in ability to understand the meaning of laboratory data |
|  |  | - Beliefs about Consequences | - Patients believe that understanding the meaning of laboratory data will be beneficial for their health outcomes |
|  |  | - Environmental context and resources | - Resources are available for patients to find information of laboratory data |
|  |  | - Social influences | - Patients received sufficient support from HCPs, families and peers. |
|  | PO.3.2.6. Patients actively understand risk factors of CKD, for instance, high blood pressure, diabetes, and medications. | - Knowledge | - Patients have sufficient knowledge about the meaning of laboratory data |
|  |  | - Social/professional role and identity | - Patients take responsibility of understanding risk factors of CKD |
|  |  | - Beliefs about capabilities | - Patients express the confidence in ability to understand risk factors of CKD |
|  |  | - Beliefs about Consequences | - Patients believe that understanding risk factors of CKD will be beneficial for their health outcomes |
|  |  | - Environmental context and resources | - Resources are available for patients to find information of risk factors of CKD |
| PO.3.3. Seeking social support (patient’s actions to seek resources or support from significant others to cope with  their disease and the negative emotions evoked by their  illness.) |  |  |  |
|  | PO.3.3.1. Patients will share experience with other patients about how to control kidney disease | - Beliefs about capabilities | - Patients express the confidence in ability to control kidney disease |
|  |  | - Optimism | - Patients express positive towards controlling kidney disease |
|  |  | - Environmental context and resources | - Resources are available for patients to share their experience |
|  | PO.3.3.2. Sharing helpless and frustrated feelings with other patients, or family or friends | - Knowledge | - Patients have sufficient knowledge about the importance of sharing feelings |
|  |  | - Skills | - Patients develop the ability to share negative feelings |
|  |  | - Environmental context and resources | - Resources are available for patients to share feelings |
|  |  | - Social influence | - Patients receive sufficient peer support, support from families and friends. |
|  | PO.3.3.3 Patients discuss with family or friends while questioning or worrying about kidney disease and solutions | - Knowledge | - Patients have sufficient knowledge about the importance of discussion with families and friends |
|  |  | - Skills | - Patients develop the ability to discuss with family or friends while questioning or worrying about kidney disease and solutions |
|  |  | - Social influence | - Patients receive sufficient peer support, support from families and friends. |
|  | PO.3.3.4 Patients tell family or friends about treatment plan, such as diet control and medication use, to get cooperation and support | - Knowledge | - Patients have sufficient knowledge about the importance of telling family or friends about treatment plan, such as diet control and medication use, to get cooperation and support |
|  |  | - Skills | - Patients develop the ability to tell family or friends about treatment plan, such as diet control and medication use, to get cooperation and support |
|  |  | - Social influences | - Patients receive sufficient support from families and friends. |
| PO.3.4. Adherence to recommended  regimens (how a patient follows the treatment regimen to control kidney disease.) |  |  |  |
|  | PO.3.4.1. Patients follow care providers’ suggestion to adjust diet habit, control weight, exercise, and choose food. | - Knowledge | - Patients have sufficient knowledge about the suggestions of adjust diet habit, control weight, exercise, and choose food. |
|  |  | - Skills | - Patients develop the ability of adjusting diet habit, control weight, exercise, and choose food, following care providers’ suggestions |
|  |  | - Social/professional role and identity | - Patients take responsibility of following care providers’ suggestion to adjust diet habit, control weight, exercise, and choose food. |
|  |  | - Beliefs about capabilities | - Patients express confidence in following care providers’ suggestion to adjust diet habit, control weight, exercise, and choose food. |
|  |  | - Beliefs about Consequences | - Patients believe that following care providers’ suggestion to adjust diet habit, control weight, exercise, and choose food will be beneficial for their health outcomes |
|  |  | - Environmental context and resources | - Health care professionals provided patients’ suggestions of adjust diet habit/educational resources, control weight, exercise, and choose food. |
| PO.4. Patients receive sufficient online support and have less face to face contacts with HCPs |  | - Knowledge | - Patients have sufficient knowledge about how to manage CKD in their daily life |
|  |  | - Skills | - Patients develop sufficient abilities to manage CKD in their daily life |
|  |  | - Environmental context and resources | - Patients receive sufficient online support to manage CKD |
|  |  | - Social influence | - Patients receive sufficient online support from HCPs |
| Interpersonal level |  |  |  |
| PO.5. Patients receive sufficient family, and peer support during process of CKD SM |  | - Knowledge | - Families and peers have sufficient knowledge about how to support patients in CKD management |
|  |  | - Skills | - Families and peers develop the ability to support patients |
|  |  | - Social/professional role and identity | - Families and peers feel it is their responsibility to support patients |
|  |  | - Beliefs about Consequences | - Families and peers believe that supporting patients will be beneficial for patients’ health outcomes and quality of life |
|  |  | - Environmental context and resources | - Educational resources or platforms are available for them to support patients |
| PO.6. Patients receive sufficient online support and have less face to face contacts with HCPs on small complaints. Therefore, HCPs can have sufficient time to focus on patients with more severe complaints. |  | - Knowledge | - Patients have sufficient knowledge about how to get online support to manage their CKD |
|  |  | - Skills | - Patients receive and integrate online support for their CKD management |
|  |  | - Environmental context and resources | - Patients can access online support to manage CKD |
|  |  | - Social influence | - Patients receive sufficient online support from HCPs |
| PO.7. Patients receive sufficient support (e.g., CKD related knowledge) from community health care center |  | - Knowledge | - HCPs in community health care centers have sufficient knowledge about about CKD management |
|  |  | - Skills | - HCPs in community health care center develop the ability in CKD management |
|  |  | - Social/professional role and identity | - HCPs in community health care center can provide sufficient support for patients with CKD |
|  |  | - Environmental context and resources | - Resources are available for HCPs in community health care center to get enough knowledge and skills in CKD management - HCPs can provide support for patients with CKD |
| Organisational |  |  |  |
| PO.8. There is less health care usage in primary and secondary care |  | - Knowledge | - Patients have sufficient knowledge about how to control CKD |
|  |  | - Skills | - Patients develop the ability in controlling CKD |
|  |  | - Social/professional role and identity | - Patients identify the responsibility of CKD management |
|  |  | - Environmental context and resources | - Patients receive sufficient resources in increasing knowledge and developing abilities |
|  |  | - Social influences | - Patients receive sufficient support from families, peers, and HCPs |
| Community |  |  |  |
| PO.9. CKD related knowledge and treatment are available in community |  | - Environmental context and resources | - HCPs in community health care centers receive sufficient educational resources/tailored trainings to support CKD self-management |
| PO.10. There is an improved collaboration between primary care and secondary care in CKD management |  | - Environmental context and resources | - The collaborations are established between primary and secondary care to support CKD management |
| Public policy |  |  |  |
| PO.11. The policy of digital health implementation in health care is more concrete |  | - Environmental context and resources | - The research provides evidence for the government to make the policy of digital health implementation in health care more concrete. |
| PO.12. There is an improved attention to digital health to improve primary care in public policy |  | - Environmental context and resources | - The research provides effectiveness of digital health |
