# Supplementary material for: Developing a Tailored eHealth Self-Management Intervention for Patients With Chronic Kidney Disease in China: Intervention Mapping Approach
Source: JMIR Form Res. 2024 Jun 13;8:e48605. doi: 10.2196/48605 (PMC11211709; doi:10.2196/48605)
Supplement: Multimedia Appendix 3 [file formative_v8i1e48605_app3.docx]

**Multimedia Appendix 3 Theoretical methods and practical strategies for changes objectives**

| **Determinant** | **Change objective** | **Patients** | **HCPs** | **Families and peers** | **Interpersonal**  **Organisational**  **Community**  **Public policy** | **Performance objective level** | **Theoretical methods** | **Practical strategy** |
| --- | --- | --- | --- | --- | --- | --- | --- | --- |
| **Knowledge**  -Know |  |  |  |  |  |  | - Feedback on behaviour - Feedback on outcome of behaviour - Information about patients of behaviour - Information about the health consequences of behaviour   (BCTS) | - Lecture - Group discussion, - Programme handbook, - Picture handout of CKD and self-management knowledge, - Local community resources handout, - Workbook |
|  | CKD related knowledge | **X** |  |  |  | **PO.2** |  |  |
|  | CKD SM related knowledge | **X** | **X** |  |  | **PO.2** |  |  |
|  | what behaviors will negatively affect kidney function, and can identify the health risk behaviors relevant to them | **X** |  |  |  | **PO.3.1.1** |  |  |
|  | portions and choices of food | **X** |  |  |  | **PO.3.1.2** |  |  |
|  | suggestions of managing food from care providers | **X** |  |  |  | **PO.3.1.3** |  |  |
|  | what health behaviours are harmful for kidney | **X** |  |  |  | **PO.3.1.4** |  |  |
|  | what CKD care and lifestyle needs to be adjusted (for instance, diet intake, physical activity, and medication use). | **X** |  |  |  | **PO.3.1.5** |  |  |
|  | the aim of managing CKD is to stay healthy | **X** |  |  |  | **PO.3.1.6** |  |  |
|  | signs and symptoms of health problems | **X** |  |  |  | **PO.3.2.3** |  |  |
|  | the meaning of laboratory data | **X** |  |  |  | **PO.3.2.4,5,6** |  |  |
|  | the importance of sharing feelings | **X** |  |  |  | **PO.3.3.2** |  |  |
|  | the importance of discussion with families and friends | **X** |  |  |  | **PO.3.3.3** |  |  |
|  | the importance of telling family or friends about treatment plan, such as diet control and medication use, to get cooperation and support | **X** |  |  |  | **PO.3.3.4** |  |  |
|  | the suggestions of adjust high-salt diet, control weight, exercise, and choose food. | **X** |  |  |  | **PO.3.4.1** |  |  |
|  | general knowledge to manage CKD in their daily life | **X** |  |  |  | **PO.4** |  |  |
|  | how to support patients in CKD management |  |  | **X** |  | **PO.5** |  |  |
|  | patients know general knowledge to manage CKD in their daily life | **X** |  |  |  | **PO.6** |  |  |
|  | the knowledge of CKD management of by HCPs in community health care center |  | **X** |  |  | **PO.7** |  |  |
|  | how to control CKD | **X** |  |  |  | **PO.8** |  |  |
| **Optimism**  -Patients are optimistic on |  |  |  |  |  |  | - Modelling | - Newsletters delivered to patients with role model stories of health care providers treating patients who had good recovery Billboards with local role models-showing good recovery - Presentations of role model stories at staff meetings |
|  | chronic kidney disease | **X** |  |  |  | **PO.1** |  |  |
|  | managing CKD | **X** |  |  |  | **PO.3.1.6** |  |  |
|  | controlling kidney disease | **X** |  |  |  | **PO.3.3.1** |  |  |
| **Beliefs about Consequences**  -Believe |  |  |  |  |  |  | - Social support—emotional - Information of emotional consequences of behaviour - Reduce negative emotions - Modelling | - Lecture - Group discussion - Social support from other participants - Feedback from HCPs, Billboards with local role models-showing good recovery |
|  | Being responsible for self-management will improve their outcomes | **X** |  |  |  | **PO.1** |  |  |
|  | optimal CKD SM improve patients’ health outcomes | **X** | **X** |  |  | **PO.2** |  |  |
|  | changing health risk behaviors will be beneficial for their health outcomes | X |  |  |  | **PO.3.1.1** |  |  |
|  | managing food portions and choices will be beneficial for their health outcomes | **X** |  |  |  | **PO.3.1.2** |  |  |
|  | manging food following care providers’ suggestions will be beneficial for their health outcomes | **X** |  |  |  | **PO.3.1.3** |  |  |
|  | giving up health risk behaviors will be beneficial for their kidney and health outcomes | **X** |  |  |  | **PO.3.1.4** |  |  |
|  | adjusting CKD care and lifestyle will be beneficial for their health outcomes | **X** |  |  |  | **PO.3.1.5** |  |  |
|  | managing CKD will be help them stay healthy | **X** |  |  |  | **PO.3.1.6** |  |  |
|  | merging CKD management into daily life will be beneficial for their health outcomes | **X** |  |  |  | **PO.3.1.7** |  |  |
|  | clarifying questions about treatment and solve problems will be beneficial for their CKD management | **X** |  |  |  | **PO.3.2.2** |  |  |
|  | finding out reasons for signs and symptoms of health problems will be beneficial for their health outcomes | **X** |  |  |  | **PO.3.2.3** |  |  |
|  | thinking over reasons about bad laboratory data will be beneficial for their health outcomes | **X** |  |  |  | **PO.3.2.4** |  |  |
|  | understanding the meaning of laboratory data will be beneficial for their health outcomes | **X** |  |  |  | **PO.3.2.5** |  |  |
|  | understanding risk factors of CKD will be beneficial for their health outcomes | **X** |  |  |  | **PO.3.2.6** |  |  |
|  | following care providers’ suggestion to adjust diet, control weight, exercise, and choose food will be beneficial for their health outcomes | **X** |  |  |  | **PO.3.4.1** |  |  |
|  | supporting patients will be beneficial for patients’ health outcomes |  |  | **X** |  | **PO.5** |  |  |
| **Environmental context and resources** |  |  |  |  |  |  | - 12.1 Restructuring the physical environment - 12.5 Adding objects to the environment | - Managers approve the redeployment/purchase of equipment to allow delivery of the eHealth intervention - Managers approve rental of leisure centre facilities to deliver the eHealth intervention if clinic space unsuitable |
|  | Educational resources and tailored training of CKD SM are available for patients and HCPs |  |  |  | **X** | **PO.2** |  |  |
|  | Educational resources, tailored training and practical applications are available for patients to manage food portions and choices  Nutritionist provide support for patients |  |  |  | **X** | **PO.3.1.2** |  |  |
|  | Suggestions of care providers are available and sufficient for patients to manage food. |  |  |  | **X** | **PO.3.1.3** |  |  |
|  | Suggestions of adjusting CKD care and lifestyle are available |  |  |  | **X** | **PO.3.1.5** |  |  |
|  | Educational resources and tailored training of managing CKD are available |  |  |  | **X** | **PO.3.1.6** |  |  |
|  | Good-quality educational resources are available for patients to seek information about kidney disease. |  |  |  | **X** | **PO.3.2.1** |  |  |
|  | Resources are available for patients to clarify questions of treatment plan and solve problems |  |  |  | **X** | **PO.3.2.2** |  |  |
|  | Resources are available for patients to find out reasons for signs and symptoms of health problems. |  |  |  | **X** | **PO.3.2.3** |  |  |
|  | Resources are available for patients to find information of laboratory data |  |  |  | **X** | **PO.3.2.4** |  |  |
|  | Resources are available for patients to find information of laboratory data |  |  |  | **X** | **PO.3.2.5** |  |  |
|  | Resources are available for patients to find information of risk factors of CKD |  |  |  | **X** | **PO.3.2.6** |  |  |
|  | Resources are available for patients to share their experience |  |  |  | **X** | **PO.3.3.1** |  |  |
|  | Resources are available for patients to share feelings |  |  |  | **X** | **PO.3.3.2** |  |  |
|  | Health care professionals provided patients’ suggestions of adjust diet/educational resources, control weight, exercise, and choose food. |  |  |  | **X** | **PO.3.4.1** |  |  |
|  | Patients receive sufficient online support to manage CKD |  |  |  | **X** | **PO.4** |  |  |
|  | Educational resources or platforms are available for them to support patients |  |  |  | **X** | **PO.5** |  |  |
|  | Patients receive sufficient online support to manage CKD |  |  |  | **X** | **PO.6** |  |  |
|  | Resources are available for HCPs in community health care center to get enough knowledge and skills in CKD management |  |  |  | **X** | **PO.7** |  |  |
|  | HCPs provide support for patients with CKD |  |  |  | **X** | **PO.7** |  |  |
|  | Patients receive sufficient resources in increasing knowledge and developing abilities |  |  |  | **X** | **PO.8** |  |  |
|  | Educational resources/tailored trainings are available in community to support CKD management |  |  |  | **X** | **PO.9** |  |  |
|  | The collaborations is established between primary and secondary care to support CKD management |  |  |  | **X** | **PO.10** |  |  |
|  | The research provides evidence for the government to make the policy of digital health implementation in health care more concrete. |  |  |  | **X** | **PO.11** |  |  |
|  | The research provides effectiveness of digital health |  |  |  | **X** | **PO.12** |  |  |
| **Skills** | Patients develop ability to manage health risk behaviors | **X** |  |  |  | **PO.3.1.1** | - Goal setting (behaviour) - Problem solving - Goal setting (outcome) - Self-monitoring of behaviour - Instruction on how to perform the behaviour - Demonstration of the behaviour - Behavioural practice/rehearsal - Generalisation of the targeted behaviour - Graded tasks - Pharmacological support - Framing/reframing - Body changes - Modelling - Skills training with guided practice and methods | - Instruction and demonstration by HCPs; - Programme handbook to record progress; feedback from HCPs; Group discussion; - Group teaching of skills by facilitators, practice by group members, identification by group members of possible negative outcomes and how to handle them - Videotaped stimulus vignettes with evaluation by participants - Skill practice in role plays with feedback - One-to-one instruction at worksites accompanied by a brochure |
|  | Patients develop the ability to manage food portions and choices | **X** |  |  |  | **PO.3.1.2** |  |  |
|  | Patients develop the ability to manage food | **X** |  |  |  | **PO.3.1.3** |  |  |
|  | Patients develop ability to give up health risk behaviors | **X** |  |  |  | **PO.3.1.4** |  |  |
|  | Patients develop ability to adjust CKD care and lifestyle. | **X** |  |  |  | **PO.3.1.5** |  |  |
|  | Patients develop ability to merge CKD management into daily life. | **X** |  |  |  | **PO.3.1.7** |  |  |
|  | Patients develop ability to seek information about kidney disease. | **X** |  |  |  | **PO.3.2.1** |  |  |
|  | Patients develop ability to clarify questions about treatment plan and solve problems | **X** |  |  |  | **PO.3.2.2** |  |  |
|  | Patients develop ability to find out reasons for signs and symptoms of health problems | **X** |  |  |  | **PO.3.2.3** |  |  |
|  | Patients develop ability to think over reasons about bad laboratory data | **X** |  |  |  | **PO.3.2.4** |  |  |
|  | Patients develop the ability to share negative feelings | **X** |  |  |  | **PO.3.3.2** |  |  |
|  | Patients develop the ability to discuss with family or friends while questioning or worrying about kidney disease and solutions | **X** |  |  |  | **PO.3.3.3** |  |  |
|  | Patients develop the ability to tell family or friends about treatment plan, such as diet control and medication use, to get cooperation and support | **X** |  |  |  | **PO.3.3.4** |  |  |
|  | Patients develop the ability of adjusting diet, control weight, exercise, and choose food, following care providers’ suggestions | **X** |  |  |  | **PO.3.4.1** |  |  |
|  | Patients develop sufficient abilities to manage CKD in their daily life | **X** |  |  |  | **PO.4** |  |  |
|  | Families and peers develop the ability in supporting patients |  |  | **X** |  | **PO.5** |  |  |
|  | Patients develop sufficient abilities to manage CKD in their daily life | **X** |  |  |  | **PO.6** |  |  |
|  | HCPs in community health care center develop the ability in CKD management |  | **X** |  |  | **PO.7** |  |  |
|  | Patients develop the ability in controlling CKD | **X** |  |  |  | **PO.8** |  |  |
| **Social/professional role and identity** | Patients take responsibility of changing health risk behaviors | **X** |  |  |  | **PO.3.1.1** | Modelling | - Newsletters delivered to patients with role model stories of health care providers treating patients who had good recovery Billboards with local role models-showing good recovery   Presentations of role model stories at staff meetings |
|  | Patients take responsibility of choosing food | **X** |  |  |  | **PO.3.1.2** |  |  |
|  | Patients take responsibility of managing food following care providers’ suggestions | **X** |  |  |  | **PO.3.1.3** |  |  |
|  | Patients take responsibility of giving up health risk behaviors | **X** |  |  |  | **PO.3.1.4** |  |  |
|  | Patients take responsibility of adjusting CKD care and lifestyle | **X** |  |  |  | **PO.3.1.5** |  |  |
|  | Patients take responsibility of clarifying questions about treatment plan and solve problems | **X** |  |  |  | **PO.3.2.2** |  |  |
|  | Patients take responsibility of finding out reasons for signs and symptoms of health problems | **X** |  |  |  | **PO.3.2.3** |  |  |
|  | Patients take responsibility of thinking over reasons about bad laboratory data | **X** |  |  |  | **PO.3.2.4** |  |  |
|  | Patients take responsibility of understanding the meaning of laboratory data | **X** |  |  |  | **PO.3.2.5** |  |  |
|  | Patients take responsibility of understanding risk factors of CKD | **X** |  |  |  | **PO.3.2.6** |  |  |
|  | Patients take responsibility of following care providers’ suggestion to adjust diet, control weight, exercise, and choose food. | **X** |  |  |  | **PO.3.4.1** |  |  |
|  | Families and peers identify the responsibility in supporting patients |  |  | **X** |  | **PO.5** |  |  |
|  | HCPs in community health care center identify the responsibility of providing sufficient support for patients with CKD |  | **X** |  |  | **PO.7** |  |  |
|  | Patients identify the responsibility of CKD management | **X** |  |  |  | **PO.8** |  |  |
| **Beliefs about capabilities** | Patients express the confidence in ability to change health risk behaviors | **X** |  |  |  | **PO.3.1.1** | - 1.2 Problem solving - 2.2 Feedback on behaviour during training - 2.3 Self-monitoring of behaviour - during training - 3.1 Social support (unspecified) - 3.2 Social support (practical) - 6.1 Demonstration of the behaviour - 8.1 Behavioural practice/rehearsal - 8.7 Graded tasks - 15.1 Verbal persuasion to boost - self-efficacy to deliver the intervention - using a needs supportive interpersonal - style - 15.3 Focus on past success - **IM**: modeling, skill - training, guided practice with feedback, and reinforcement. (P318) | - Workshop - Verbal feedback by research team during training; - Role play of delivering the intervention using a   needs supportive interpersonal style.   - Verbal and written feedback of training - Audio-recording of delivering the intervention - Using a needs supportive interpersonal style by   research team following training  Audio-recording of delivery of components of the intervention during training and self-rating of quality of delivery using a needs supportive   - Interpersonal style following training - Encouragement from facilitator and peers for - delivery of the intervention as intended during role play in training - Practical support from facilitator in supporting delivery of the intervention following training - Group discussion, program handbook to review process, support from other participants, practice by participant, feedback from health care providers - Workshop, verbal feedback, audio-recording of delivery of intervention - Encouragement from facilitator and peers for   delivery of the intervention as intended during   - Role play in training |
|  | Patients express the confidence in ability to manage food portions and choices. | **X** |  |  |  | **PO.3.1.2** |  |  |
|  | Patients express the confidence in ability to manage food following care providers’ suggestions | **X** |  |  |  | **PO.3.1.3** |  |  |
|  | Patients express the confidence in ability to give up health risk behaivors | **X** |  |  |  | **PO.3.1.4** |  |  |
|  | Patients express the confidence in ability to adjust CKD care and lifestyle | **X** |  |  |  | **PO.3.1.5** |  |  |
|  | Patients express the confidence in ability to find out reasons for signs and symptoms of health problems | **X** |  |  |  | **PO.3.2.3** |  |  |
|  | Patients express the confidence in ability to think over reasons about bad laboratory data | **X** |  |  |  | **PO.3.2.4** |  |  |
|  | Patients express the confidence in ability to understand the meaning of laboratory data | **X** |  |  |  | **PO.3.2.5** |  |  |
|  | Patients express the confidence in ability to understand risk factors of CKD | **X** |  |  |  | **PO.3.2.6** |  |  |
|  | Patients express the confidence in ability to control kidney disease | **X** |  |  |  | **PO.3.3.1** |  |  |
|  | Patients express confidence in following care providers’ suggestion to adjust diet, control weight, exercise, and choose food. | **X** |  |  |  | **PO.3.4.1** |  |  |
| - **Intentions** | Patients have the intention to change health risk behaviors. | **X** |  |  |  | **PO.3.1.1** | - 1.8 Behavioural contract | - Lecture - Group discussion - Social support from other participants - Programme handbook to record physical activity goal; - Feedback from physiotherapist |
| - **Behavioural regulation** | A patient identify strategies to break their previous health risk behaviors | **X** |  |  |  | **PO.3.1.1** | - Self-monitoring of behaviour | - Programme handbook to record progress |
|  | A patient identify strategies to manage food portions and choices. | **X** |  |  |  | **PO.3.1.2** |  |  |
|  | A patient identify strategies to manage food. | **X** |  |  |  | **PO.3.1.3** |  |  |
|  | A patient identify strategies to give up health risk behaviors | **X** |  |  |  | **PO.3.1.4** |  |  |
|  | A patient identify strategies to adjust CKD care and lifestyle | **X** |  |  |  | **PO.3.1.5** |  |  |
| - **Social influence** | Patients received sufficient support from HCPs, families and peers. | **X** |  |  |  | **PO.3.2.3** | - Restructuring the social environment | - To ensure consistency across sites in delivery   of the intervention  one trained   - Family and HCP support |
|  | Patients received sufficient support from HCPs, families and peers. | **X** |  |  |  | **PO.3.2.4** |  |  |
|  | Patients received sufficient support from HCPs, families and peers. | **X** |  |  |  | **PO.3.2.5** |  |  |
|  | Patients receive sufficient peer support, support from families and friends. | **X** |  |  |  | **PO.3.3.2** |  |  |
|  | Patients receive sufficient peer support, support from families and friends. | **X** |  |  |  | **PO.3.3.3** |  |  |
|  | Patients receive sufficient support from families and friends. | **X** |  |  |  | **PO.3.3.4** |  |  |
|  | Patients receive sufficient online support from HCPs | **X** |  |  |  | **PO.4** |  |  |
|  | Patients receive sufficient online support from HCPs | **X** |  |  |  | **PO.6** |  |  |
|  | Patients receive sufficient support from families, peers, and HCPs | **X** |  |  |  | **PO.8** |  |  |
