# Supplementary material for: Developing a Tailored eHealth Self-Management Intervention for Patients With Chronic Kidney Disease in China: Intervention Mapping Approach
Source: JMIR Form Res. 2024 Jun 13;8:e48605. doi: 10.2196/48605 (PMC11211709; doi:10.2196/48605)
Supplement: Multimedia Appendix 4 [file formative_v8i1e48605_app4.docx]

**Multimedia Appendix 4** **Guiding principles for a tailored “MD”-based intervention (plan) to improve self-management in patients with chronic kidney disease**

The tailored ‘MD’ based intervention should

- meet individual patient needs, perceptions, and preferences regarding CKD self-management
- focus on the knowledge of, and motivation and skills towards CKD self-management
- be safe, literacy-sensitive, and user-friendly
- be feasible for the current Chinese CKD care
- develop a trustworthy health education resource platform on CKD (self-management)
- include adequate family-level support and effective patients–HCP communication
- fit well with and supports HCPs’ clinical workflows
- be delivered via mobile phone
- make use of the paternalistic guidance by HCPs to help build a strong belief on importance and potential benefits of self-management
