# Supplementary material for: Developing a Tailored eHealth Self-Management Intervention for Patients With Chronic Kidney Disease in China: Intervention Mapping Approach
Source: JMIR Form Res. 2024 Jun 13;8:e48605. doi: 10.2196/48605 (PMC11211709; doi:10.2196/48605)
Supplement: Multimedia Appendix 5 [file formative_v8i1e48605_app5.docx]

**Multimedia Appendix 5. Logic model definitions and examples for a tailored “medical dashboard”–based self-management intervention for patients with chronic kidney disease**

| **Inputs** | **Outputs** | | **Short-term**  **outcomes** | **Medium-outcomes** | **Long-term**  **outcomes** |
| --- | --- | --- | --- | --- | --- |
|  | *Who we reach* | *What we do* |  |  |  |
| Resources include human, financial,  organizational, and  community resources  available to direct  toward doing the activity. | Individuals targeted by the activity. They may be the recipients of an activity but are not necessarily the recipients of the program. | The “interventions” that produce the direct products. | Outcomes examine changes directly connected to intervention implementation, typically including knowledge, skills, or attitudes contributing to medium outcomes. These are the prerequisite changes expected as a result of the intervention implementation. | Outcomes are specific, measurable changes in things like behavior change, decision-making practices, and community resources acting as a bridge between short- and long-term outcomes. | Outcomes are ultimate changes or impacts, typically including improved health behavior, health conditions, increased capacity, and/or changes in programmatic reach. |
| ***examples****:*   - *staff* - *funding* | ***examples****:*   - *Patients* - *HCPs* - *Primary and secondary care professionals* - *Community* | ***examples:***   - *Workshop on* - *Materials development* | ***examples****:*   - *Increased basic knowledge of self-management such as core principles, application, and benefits of self-management, the importance of becoming an active self-manager, taking actions, and forming an action plan.* - *Improved skills of intervention use, such as monitoring the blood pressure, measure sodium intake using the eHealth program* | ***examples:***   - *Participants improved the knowledge to self-management behavior needed to manage symptoms and illness as well as to improve compliance to treatment and promoting a healthy lifestyle.* - *Participants improved the CKD self-management skills performed for health behaviour change or decision making practice such as adhering to low-salt intake diet* | ***examples:***   - *Biomedical status (e.g., CKD progression measured with an estimated glomerular filtration rate and serum creatinine) and patient safety (adverse events)* - *Self-efficacy* - *Quality of life* |
