# Supplementary material for: Developing a Tailored eHealth Self-Management Intervention for Patients With Chronic Kidney Disease in China: Intervention Mapping Approach
Source: JMIR Form Res. 2024 Jun 13;8:e48605. doi: 10.2196/48605 (PMC11211709; doi:10.2196/48605)
Supplement: Multimedia Appendix 6 [file formative_v8i1e48605_app6.docx]

**Multimedia Appendix 6 Core intervention components and functionalities of the medical dashboard**

- **Motivational interviewing:** Patients are provided with a one-hour individual motivational interview, which focuses on discussing barriers, benefits, and strategies for self-management; setting personal goals, and strengthening intrinsic motivation and self-efficacy.
- **Education:** Patients are provided with education, a kidney-friendly cookbook, instructions for self-monitoring blood pressure (using a Microlife Watch blood pressure home device), dietary intake (using an online food diary) and 24-hour urinary sodium excretion (using an innovative point-of-care chip device).
- **Self-monitoring**: Patients are instructed to take health measurements at home (e.g. blood pressure, weight and glucose) and enter the results of these measurements via the secure “self-care” website [www.bonstat.nl](http://www.bonstat.nl). The measurements entered via this website are linked real-time to the Medical Dashboard interface.
- **Combination of home and hospital measurements in the Medical Dashboard**: The measurements that patients take at home and the measurements performed during hospital visits are visualized jointly in the Medical Dashboard.
- **Online information support**: Patients are provided with online disease-related information, tips and suggestions focusing not only on medical knowledge, but also on how to obtain and sustain social support, refusal skills, medication adherence strategies, physical exercise, healthy eating, smoking cessation and reduced alcohol intake.
- **Personal coaching**: Patients are coupled with one of four personal coaches: three health psychologists and one dietician. Following the self-monitoring measurements, patients are provided with feedback by telephone from their coach or during hospital visits. The discussion focuses on the progression, achievements, barriers and possible solutions of self-management.

A


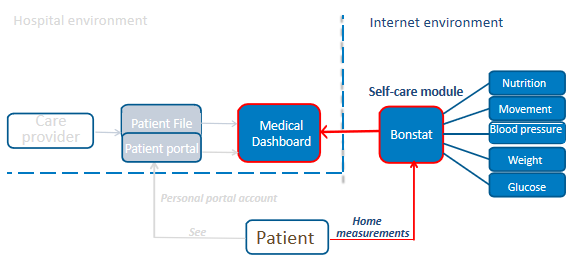


B


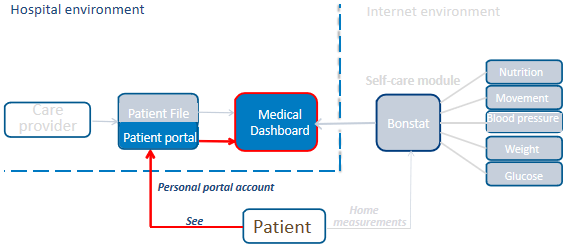


C


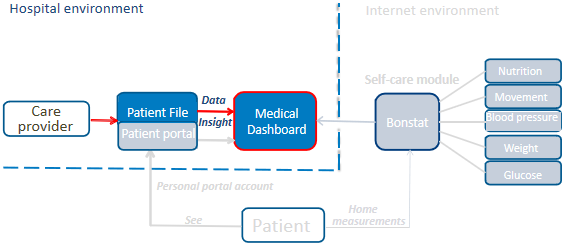


Medical Dashboard. (A) self-monitoring; (B, C) combination of home and hospital measurements in the Medical Dashboard, online information support
