# Supplementary material for: Developing a Tailored eHealth Self-Management Intervention for Patients With Chronic Kidney Disease in China: Intervention Mapping Approach
Source: JMIR Form Res. 2024 Jun 13;8:e48605. doi: 10.2196/48605 (PMC11211709; doi:10.2196/48605)
Supplement: Multimedia Appendix 7 [file formative_v8i1e48605_app7.docx]

**Multimedia Appendix 7 A summary of intervention adaptations and priorities of eHealth features**

| **Original MD intervention**  **component** | **Rationale of adaptation** | **Description of the adapted intervention component** |
| --- | --- | --- |
| **Education** | - The educational materials and instructions for eHealth use need to be available in Chinese and include relatable, engaging, and relevant formats to Chinese patients. - Considering some vulnerable groups' low health literacy level, face-to-face methods for personal assistance and coaching should be used. - The need for tailored education and training for patients and HCPs on the core concepts and possible advantages of self-management interventions before intervention implementation was highlighted. | - **Education**   Patients are provided with self-management education, a kidney-friendly cookbook, eHealth handbook with instructions of eHealth use such as self-monitoring blood pressure, dietary intake and 24-hour urinary sodium excretion.  Blended learning that combines e-learning and face-to-face methods supports self-management education development for patients and HCPs. For instance, lectures, group discussions, and community resources handouts of self-management and eHealth use are held. Educational activities of eHealth simulation exercises and real-life practice are also provided. |
| **Motivational interviewing** | - Lack of knowledge is a barrier that negatively impacts patients’ optimism, beliefs about consequences, and their involvement in their self-management (skills development) in China. - Making use of the paternalistic guidance by HCPs to help build a firm belief in the importance and potential benefits of self-management - The psychosocial burden that patients face in their everyday lives can be overwhelming. Therefore, a more individualized and specialized empowerment intervention is needed. | - **Motivational interviewing**   Patients are provided with a one-hour individual motivational interview, which focuses on discussing barriers, benefits, and strategies for self-management; setting personal goals, and strengthening intrinsic motivation and self-efficacy. Also, this interview helps to train patients’ skills in posing more and better self-management related questions to their doctors, such as including a patient activation checklist to help patients prepare for their visits with HCPs and be more active during these visits.   - **Modeling**   Community health care centers deliver newsletters to patients with role model stories of HCPs helping patients’ self-management who had a good recovery. Presentations of role model stories at lectures and group discussions are provided. |
| **Self-monitoring** | - Patients and HCPs mentioned that the computer-based version of MD was challenging to use as it was different from patients’ previous experience with eHealth technology (i.e. smartphone). For instance, the need to log in to the MD via a separate website was mentioned. Also, China has 1.3 billion mobile phone users (penetration rate of 90%), it is necessary to extend the intervention delivery medium to a mobile phone app, combined with a wireless tracker to collect measurements automatically. Additionally, patients expressed the need of contacting HCPs when their parameters are abnormal. | - **Self-monitoring**   patients are instructed to take health measurements at home (e.g. blood pressure, weight, and glucose) and enter the results of these measurements via the secure “patient self-care” mobile phone app.  The measurements entered via this patient self care are linked in real-time to the HCPs interface in this app and the progress is recorded. Reminders are sent to HCPs when patients-entered data is abnormal and (online) communications are provided |
| **Combination of home and hospital measurements in the MD** | - Patients and HCPs mentioned that the computer-based version of MD was challenging to use as it was different from their previous experience with eHealth technology (i.e. smartphone). | - **Combination of home and hospital measurements**   The measurements patients take at home and those performed during hospital visits are visualized jointly in the mobile health application. |
| **Online information support** | - The online disease-related information, tips and suggestions need to be available in Chinese and adaptations of educational self-management materials, such as informative videos, are needed to ensure that these materials are relevant to individual patients’ daily life in Chinese settings. - Considering some vulnerable groups' low health literacy level, face-to-face methods for personal assistance and coaching should be used. - A smartphone-based online information support is needed. - Patients with CKD expressed a need for an online information platform established by the government or hospital. | - **Information support (online and offline)** - Patients are provided with chatbot-based support including online disease-related information, tips and suggestions focusing not only on medical knowledge, but also on how to obtain and sustain social support, refusal skills, medication adherence strategies, physical exercise, healthy eating, smoking cessation, and reduced alcohol intake. - Lectures by HCPs, group teaching, and community resources handout are held. |
| **Personal coaching** | - In comparison to most western countries, Chinese society is more collective, and good relations with family members and peers in the patient's community are of utmost importance. Therefore, for instance, to change patients’ health risk behaviors of high-salt eating, caregivers need to be informed, and in agreement and ensure family meals meet the requirements of a low-salt diet. | **Coaching (Individual and group coaching)**  Patients and their families are coupled with one of four personal coaches: three health psychologists and one dietician. Following the self-monitoring measurements, they are provided with feedback by telephone from their coach or during hospital visits. The discussion focuses on the progression, achievements, barriers and possible self-management solutions.  Identification by group members of possible adverse outcomes and how to handle them, videotaped stimulus vignettes with evaluation by participants, skill practice in role plays with feedback, and one-to-one instruction at worksites accompanied by a brochure are provided. |
