# Supplementary material for: Developing a Tailored eHealth Self-Management Intervention for Patients With Chronic Kidney Disease in China: Intervention Mapping Approach
Source: JMIR Form Res. 2024 Jun 13;8:e48605. doi: 10.2196/48605 (PMC11211709; doi:10.2196/48605)
Supplement: Multimedia Appendix 8 [file formative_v8i1e48605_app8.docx]

**Multimedia Appendix 8 Consolidated Framework for Implementation Research (CFIR)–Expert Recommendations for Implementing Change Matching Tool for strategies across all CFIR determinants**

| **ERIC Strategies** | **Cumu-lative Perce-nt** | **Evid-ence Stre-ngth & Qual-ity** | **Relat-ive adva-ntage** | **Comp-lexity** | **Patie-nt Needs & Reso-urces** | **Structural Character-istics** | **Cul-ture** | **Compat-ibility** | **Avail-able Reso-urces** | **Access to knowl-edge & inform-ation** | **Knowl-edge & Beliefs about the Interve-ntion** | **Self-effic-acy** | **Indivi-dual Stage of Chan-ge** | **Plan-**  **ning** | **Exec-**  **uting** |
| --- | --- | --- | --- | --- | --- | --- | --- | --- | --- | --- | --- | --- | --- | --- | --- |
| Identify and prepare champions | **407%** | **X** | **X** | **X** |  | **X** | **X** | **X** |  | **X** | **X** | **X** | **X** | **X** |  |
| Assess for readiness and identify barriers and facilitators | **348%** |  | **X** | **X** | **X** | **X** | **X** | **X** |  |  | **X** |  |  | **X** | **X** |
| Conduct educational meetings | **312%** | **X** | **X** |  |  |  | **X** |  |  | **X** | **X** |  | **X** |  |  |
| Capture and share local knowledge | **274%** | **X** |  | **X** |  | **X** | **X** |  | **X** | **X** | **X** |  |  |  |  |
| Create a learning collaborative | **273%** |  |  | **X** |  |  | **X** |  |  | **X** |  | **X** | **X** |  | **X** |
| Conduct local consensus discussions | **250%** | **X** | **X** |  | **X** |  | **X** | **X** |  |  |  |  | **X** | **X** |  |
| Promote adaptability | **248%** |  | **X** | **X** |  | **X** | **X** | **X** |  |  |  |  | **X** |  |  |
| Conduct local needs assessment | **240%** |  | **X** |  | **X** |  | **X** | **X** |  |  | **X** |  |  | **X** |  |
| Develop a formal implementation blueprint | **222%** |  |  | **X** |  |  |  |  |  |  |  |  |  | **X** | 28% |
| Conduct cyclical small tests of change | **222%** |  | **X** | **X** |  | **X** |  | **X** |  |  |  | **X** |  |  |  |
| Develop educational materials | **220%** | **X** |  |  |  |  |  |  |  | **X** | **X** |  | **X** |  |  |
| Conduct ongoing training | **220%** |  |  | **X** |  |  |  |  |  | **X** |  | **X** | **X** | **X** | **X** |
| Tailor strategies | **212%** |  |  | **X** |  |  | **X** | **X** |  |  |  |  |  |  |  |
| Facilitation | **205%** |  |  | **X** |  |  | **X** | **X** |  |  | **X** | **X** |  | **X** | **X** |
| Identify early adopters | **201%** | **X** |  | **X** |  | **X** |  |  |  |  | **X** |  | **X** |  |  |
| Alter incentive/allowance structures | **188%** |  | **X** |  |  |  |  |  |  |  |  |  | **X** |  |  |
| Inform local opinion leaders | **188%** | **X** | **X** |  |  |  | **X** |  |  |  | **X** |  | **X** |  |  |
| Conduct educational outreach visits | **183%** | **X** |  |  |  |  |  |  |  | **X** | **X** | **X** | **X** |  |  |
| Provide ongoing consultation | **171%** |  |  | **X** |  |  |  |  |  |  |  | **X** |  |  | **X** |
| Provide local technical assistance | **165%** |  |  |  |  |  |  |  |  | **X** |  | **X** |  |  | **X** |
| Stage implementation scale up | **160%** |  |  | **X** |  |  |  |  |  |  | **X** |  |  |  |  |
| Build a coalition | **157%** |  |  |  |  | **X** |  | **X** |  |  |  |  |  |  |  |
| Organize clinician implementation team meetings | **157%** |  |  | **X** |  |  |  |  |  |  |  |  |  |  | **X** |
| Distribute educational materials | **147%** | **X** |  |  |  |  |  |  |  | **X** |  |  |  |  |  |
| Visit other sites | **145%** |  | **X** |  |  |  |  |  |  |  |  |  |  |  |  |
| Involve patients/consumers and family members | **144%** |  |  |  | **X** |  |  |  |  |  |  |  |  |  |  |
| Purposely reexamine the implementation | **142%** |  |  |  |  |  |  | **X** |  |  |  |  |  |  | **X** |
| Model and simulate change | **138%** |  |  | **X** |  |  |  |  |  |  |  | **X** |  |  |  |
| Obtain and use patients/consumers and family feedback | **131%** |  |  |  | **X** |  |  |  |  |  |  |  |  |  |  |
| Use advisory boards and workgroups | **128%** |  |  |  | **X** |  | **X** |  |  |  |  |  |  |  |  |
| Access new funding | **122%** |  |  |  |  |  |  |  | **X** |  |  |  |  |  |  |
| Use an implementation adviser | **121%** |  |  |  |  |  |  |  |  |  |  |  |  |  | **X** |
| Develop and implement tools for quality monitoring | **119%** |  |  |  |  |  |  |  |  |  |  |  |  | **X** | **X** |
| Make training dynamic | **119%** |  |  |  |  |  |  |  |  |  |  | **X** | **X** |  |  |
| Fund and contract for clinical innovation | **107%** |  |  |  |  |  |  |  | **X** |  |  |  |  |  |  |
| Recruit, designate and train for leadership | **103%** |  |  |  |  |  | **X** |  |  |  |  |  |  |  |  |
| Audit and provide feedback | **101%** |  |  |  |  |  |  |  |  |  |  | **X** |  |  |  |
| Change physical structure and equipment | **97%** |  |  |  |  | **X** |  |  | **X** |  |  |  |  |  |  |
| Shadow other experts | **95%** |  |  |  |  |  |  |  |  | **X** |  | **X** |  |  |  |
| Facilitate relay of clinical data to providers | **92%** |  |  |  |  |  |  |  |  |  |  |  |  |  |  |
| Develop academic partnerships | **88%** | **X** |  |  |  |  |  |  |  |  |  |  |  |  |  |
| Promote network weaving | **80%** |  |  |  |  | **X** |  |  |  |  |  |  |  |  |  |
| Involve executive boards | **74%** |  |  |  |  |  |  |  |  |  |  |  |  |  |  |
| Increase demand | **73%** |  | **X** |  |  |  |  |  |  |  | **X** |  |  |  |  |
| Provide clinical supervision | **68%** |  |  |  |  |  |  |  |  |  |  |  |  |  |  |
| Develop and organize quality monitoring systems | **68%** |  |  |  |  |  |  |  |  |  |  |  |  |  | **X** |
| Centralize technical assistance | **65%** |  |  |  |  |  |  |  |  |  |  |  |  |  |  |
| Intervene with patients/consumers to enhance uptake & adherence | **60%** |  |  |  | **X** |  |  |  |  |  |  |  |  |  |  |
| Use train the trainer strategies | **59%** |  |  |  |  |  |  |  |  |  |  |  |  |  |  |
| Prepare patients/consumers to be active participants | **58%** |  |  |  | **X** |  |  |  |  |  |  |  |  |  |  |
| Create new clinical teams | **58%** |  |  |  |  |  |  |  |  |  |  |  |  |  |  |
| Develop resource sharing agreements | **56%** |  |  |  |  |  |  |  | **X** |  |  |  |  |  |  |
| Revise professional roles | **55%** |  |  |  |  |  |  |  |  |  |  |  |  |  |  |
| Mandate change | **52%** |  |  |  |  |  |  |  |  |  |  |  |  |  |  |
| Obtain formal commitments | **50%** |  |  |  |  |  |  |  |  |  |  |  |  |  |  |
